# Pharmacological HIF activation protects against diet-induced obesity, glucose intolerance, and skeletal dysfunction by exerting dual beneficial effects on energy metabolism and bone

**DOI:** 10.1038/s41413-025-00503-3

**Published:** 2026-02-11

**Authors:** Roger Valle-Tenney, Nicolas Peredo, Karen De Samblancx, Elena Nefyodova, Ruben Cardoen, Tom Dehaemers, Delphine Farlay, Roland Chapurlat, Bart Van der Schueren, Chantal Mathieu, Roman Vangoitsenhoven, Christa Maes

**Affiliations:** 1 Laboratory of Skeletal Cell Biology and Physiology (SCEBP), Skeletal Biology and Engineering Research Center (SBE), Department of Development and Regeneration, KU Leuven, Leuven, Belgium; 2 INSERM, UMR 1033, Univ Lyon, Université Claude Bernard Lyon 1, Lyon, France; 3 Department of Endocrinology, University Hospitals Leuven, and Department of Chronic Diseases and Metabolism, KU Leuven, Leuven, Belgium; 4 Present Address: VIB BioImaging Core Leuven, Center for Brain and Disease Research, KU Leuven, Leuven, Belgium

**Keywords:** Pre-diabetes, Obesity, Obesity, Metabolic bone disease, Bone

## Abstract

Obesity and type-2 diabetes, two interconnected and increasingly prevalent metabolic disorders, are associated with poor bone quality, higher fracture risk, and impaired fracture repair. The causes are not yet resolved but appear to relate to the impaired glucose homeostasis, altered bone material properties and remodeling, and compromised skeletal vascularization. Each of these features is impacted by hypoxia-inducible factor (HIF) signaling, which led us to hypothesize that HIF pathway modulation might be an effective strategy to concomitantly improve energy metabolism and bone health in conditions of metabolic stress. Here, we evaluated whether pharmacological HIF activation using the HIF-prolyl-hydroxylase-domain enzyme (PHD) inhibitor FG-4592 (Roxadustat) could protect mice against the adverse skeletal and metabolic consequences of high-fat diet (HFD)-induced obesity. We found that systemic FG-4592 treatment effectively prevented HFD-triggered body weight gain, glucose intolerance, and peripheral fat accumulation, associated with globally increased energy expenditure. Concomitantly, FG-4592 administration prevented the skeletal vascular damage, marrow fat accumulation, and bone formation deficits that were caused by HFD. Moreover, the HIF-activating drug also improved glucose metabolism and bone regeneration in a model of compromised fracture repair associated with overnutrition. Specifically, short-term FG-4592 treatment during fracture recovery reduced the body weight and fat mass of obese mice, improved glucose tolerance, and enhanced the fracture bridging capacity, along with promoting callus vascularization. These findings demonstrate that systemic hypoxia signaling stimulation using PHD inhibitors alleviates both the metabolic and skeletal consequences of diet-induced obesity in mice, highlighting its potential as a dual-action therapeutic strategy for enhancing glucose homeostasis and bone health/regeneration in disorders of obesity and metabolic dysfunction.

## Introduction

Obesity is a chronic disease characterized by an excessive accumulation of body fat, predisposing affected individuals to various life-threatening health complications, including cardiovascular diseases, certain cancers, liver diseases, bone and joint problems, and reproductive and hormonal complications.^1,2^ Moreover, obesity is a leading cause of metabolic syndrome and the most critical and significant risk factor for developing insulin resistance and type-2 diabetes mellitus (T2DM).^2–4^ With the prevalence of obesity rising at an alarming rate, a concomitant rapid increase is occurring in the incidence of T2DM, which is associated with multiple harmful consequences, including inflammation and adipose tissue dysfunction, ectopic fat distribution, declined pancreatic β-cell function affecting insulin production, and multi-organ insulin resistance.^3,4^ According to the World Health Organization, in 2022, approximately 1 in 8 people worldwide were living with obesity, defined as a body mass index equal to or higher than 30, forming a global epidemic affecting >890 million adults and even over one billion people, considering all aged >5 years.^5^

Both obesity and T2DM have been associated with reduced bone quality, increased fracture risk, and impaired bone healing.^6–11^ The link between obesity and fracture risk is complex and depends on multiple factors, such as sex, age, comorbidities, and skeletal site.^12^ As such, bone fragility in people with obesity seems to be most commonly localized to the lower limbs, proximal humerus, and ankle, whereas fractures to the hip, wrist and pelvis are less prevalent.^6–9^ Most characteristically, the increased skeletal fragility in these patients presents as independent from bone mineral density (BMD), which is often normal or even higher than normal, allegedly due to the impact of the increased mechanical load on the bone, in combination with elevated estrogen production in the expanded adipose tissues.^7^ This observation, also known as the obesity paradox, underscores that the key problem is not a reduced quantity of bone but rather an impaired quality, suggesting that changes in the bone microenvironment are critical in the pathophysiology of metabolic disorders-related bone disease. Both obesity and diabetes are commonly associated with increased oxidative stress and a chronic low-grade pro-inflammatory state, which negatively influences the skeletal environment and disrupts the bone remodeling balance.^8^ Moreover, obesity and T2DM are both associated with fracture healing complications and an increased risk of non-unions in humans,^10,11,13^ further pointing to the impact of altered environmental cues and cellular-molecular responses.

Notwithstanding differences in the severity and specifics, the pathophysiology underlying the increased bone fragility and compromised fracture repair in obesity and T2DM has many aspects in common.^7^ Mechanistically, human studies and mouse models of overnutrition with or without overt symptoms of diabetes have implicated negative effects on bone mineralization and material properties,^14,15^ bone vascularization, pericytes, and skeletal stem/progenitor cell (SSPC) function,^16–18^ along with increased adiposity in the bone marrow (BM) and callus,^9,19–22^ and cellular senescence.^14,19,23^ Hyperlipidemia and hyperglycemia, typically accompanying metabolic dysfunction disorders, impair osteoblast differentiation and favor adipogenesis from SSPCs, adding to the expansion of the bone marrow adipose tissue (BMAT).^9,18,24–26^ Features that may be more prominently detrimental in T2DM include manifest or prolonged hyperglycemia, which promotes the accumulation of advanced glycation end-products (AGEs) that are harmful for bone matrix maturation and lead to impaired bone material properties, osteogenesis, and fracture regeneration.^14,27–29^ Secondly, T2DM is well-known to lead to blood vessel deterioration and macro- and microvascular complications, compromised blood perfusion, and tissue hypoxia.^8,11,30^ Microvascular disease could plausibly also occur in bone,^11,16^ and explain, among other defects, the impaired bone formation and increased cortical porosity in T2DM.^18,27^ Overall, the mechanisms by which obesogenic and diabetic conditions affect bone quality and fracture recovery are clearly multifactorial, but at present insufficiently characterized. A better understanding of the impact of metabolic stress on the BM microenvironment, particularly bone vascularity and adiposity, could open targeted treatment modalities for this growing group of patients, which currently are lacking.^27,31,32^

Interestingly, the activation of the cells’ protective responses to hypoxia through the hypoxia-inducible factor (HIF) is often abnormal in metabolically challenged circumstances, which can result in various modes of cellular dysfunction.^33^ The hypoxia signaling pathway, and its principal transcriptional mediators HIF-1α and HIF-2α, normally controls the cellular response to hypoxia, by stimulating tissue vascularization and promoting oxygen delivery through erythropoiesis on the one hand, and by triggering a metabolic switch to oxygen-sparing glycolysis on the other.^34,35^ This switch away from oxidative metabolism is mediated by HIF-induced expression of glucose transporters and glycolytic enzymes, and allows cells to adapt to and survive in low oxygen concentrations.^34,35^ In the skeleton, adequate vascularization and oxygen supply are critical for bone formation and remodeling,^36,37^ and HIF-functions have been amply implicated in fetal skeletogenesis, postnatal bone homeostasis, age-related bone maintenance, and fracture repair.^38,39^ During endochondral bone development, HIF-1α is required to support chondrocyte survival in the hypoxic growth plate,^40,41^ and to couple angiogenesis to bone formation through its key downstream effector vascular endothelial growth factor (VEGF).^36,38,42–44^ In adulthood, signaling through HIFs is essential for bone mass acquisition and maintenance, and for fracture repair, as shown using genetic loss-of-function mouse models.^45–48^

During normal physiology, the activity of HIF is tightly controlled by the oxygen availability. In normoxia, oxygen-sensing HIF-prolyl hydroxylase domain-containing enzymes (PHDs) mediate the degradation of HIF-α by hydroxylating specific proline residues in the protein, thereby marking it for ubiquitination by the ubiquitin E3 ligase Von Hippel Lindau (VHL) and subsequent proteasomal degradation.^34,35^ Genetic activation of HIF signaling in osteolineage cells, by deleting VHL or PHDs, has been amply shown to strongly induce osteogenesis, angiogenesis, and glycolysis, resulting in increased bone mass and vascularization, and improved fracture repair.^45,46,49–51^ Moreover, use of the HIF-PHD-inhibitory drug FG-4592 or the α-ketoglutarate analog dimethyloxalylglycine (DMOG) to inhibit PHD functioning was partially protective against bone loss following ovariectomy in mice and rats, by enhancing osteogenesis and angiogenesis.^52,53^ Also, local administration of DMOG or the iron chelator and hypoxia-mimetic deferoxamine (DFO) at the site of a fracture or distraction osteogenesis procedure in the mouse femur improved bone formation and neovascularization in the callus.^46,54,55^ Thus, substantial research highlighted the potential therapeutic benefit of HIF signaling manipulation to promote bone health and regeneration, and several preclinical studies reported encouraging results on the use of pharmacological hypoxia signaling pathway modulators for these purposes. Yet, information in settings related to obesity or T2DM is lacking.

Interestingly, though, there is evidence suggesting that HIF signaling activation could have beneficial effects on energy metabolism too, even though the impact of HIF modulation in various tissues and organs is complex and incompletely understood.^56^ In our own work, for instance, we found that HIF activation by conditional deletion of *Vhl* in bone cells not only led to local effects of increased bone mass, hypervascularization and enhanced glycolysis, but also to an overall increase in glucose uptake by the skeleton and a concomitant improvement of systemic glucose homeostasis in the mice, resulting in lowered glycemia and a lean phenotype.^49,57^ Moreover, another study suggested that FG-4497, a small molecule inhibitor of HIF-PHDs, could improve glucose and lipid metabolism and protect against high-fat diet (HFD)-induced obesity in mice.^58^ Thus, HIF modulation could offer therapeutic opportunities for ameliorating metabolic symptoms, which are increasingly important to be explored in the framework of obesity-related disorders.^56^

Taken altogether, these preceding findings led us to hypothesize that pharmacological activation of the hypoxia signaling pathway through the administration of HIF-PHD inhibitors could have combined beneficial effects, both on systemic energy metabolism and on bone health and regeneration. Such dual treatment advantage could be particularly valuable and synergistic in the context of bone disease and regenerative insufficiency associated with obesogenic and/or (pre-)diabetic conditions. Therefore, we here evaluated the impact of systemic pharmacological HIF activation on glucose metabolism as well as bone homeostasis and fracture regeneration in a mouse model of HFD-induced obesity and metabolic dysfunction. We found that administration of a HIF-PHD inhibitor protected against body weight gain, fat accumulation, and glucose intolerance, and prevented the adverse impacts of HFD that were observed on bone formation, BM adiposity, and skeletal vascularization. Moreover, the treatment effectively restored the metabolically compromised fracture repair under HFD. These findings show that, at least in mice, HIF-PHD inhibition can alleviate both the metabolic and skeletal consequences of overnutrition.

## Results

### Short-term pharmacological activation of the hypoxia signaling pathway increases trabecular bone mass and skeletal vascularization

**Fig. 1:**
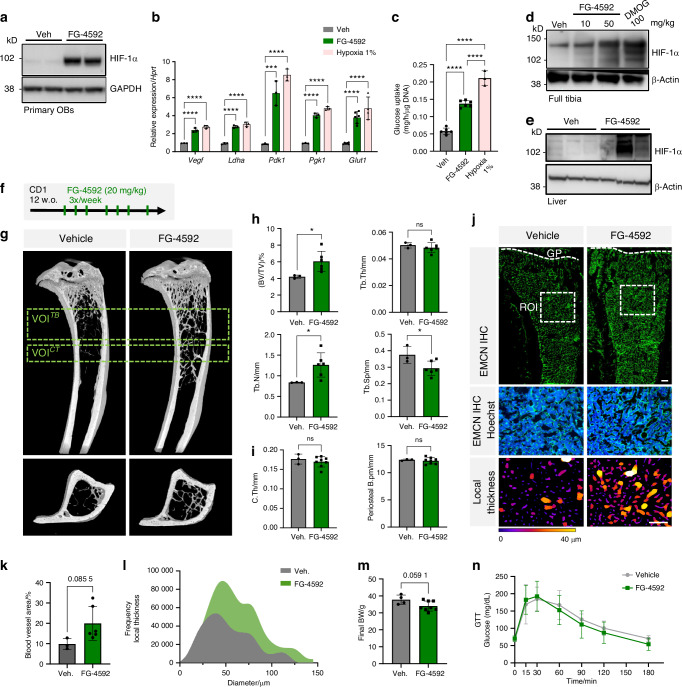
Short-term systemic FG-4592 administration activates the hypoxia signaling pathway and increases bone mass and vascularization. **a** Western blot showing HIF-1α stabilization in primary osteoblasts (pOBs) treated with vehicle or FG-4592 (20 μmol/L) for 2 h (*n* = 2), including GAPDH as loading control. **b** qRT-PCR of HIF target genes in pOBs treated with FG-4592 (20 μmol/L) for 24 h, using hypoxia incubation (1% O_2_) as positive control and *Hprt* as housekeeping gene (*n* = 3). **c** Glucose consumption (mg/h/µg DNA) of pOBs treated with vehicle or FG-4592 (20 μmol/L) for 24 h (*n* = 6), with hypoxia as positive control (*n* = 3). **d** Western blot for HIF-1α on tibia extracts of 12-week-old CD1 mice treated with vehicle, 10 or 50 mg/kg FG-4592 i.p. for 3 h, or DMOG 100 mg/kg (as positive control). β-actin, loading control. **e** Western blot for HIF-1α on liver extracts of 12-week-old CD1 mice treated with FG-4592 (20 mg/kg i.p.) for 6 h, each lane representing a different animal. **f** Short-term FG-4592 treatment scheme applied to 12-week-old male CD1 mice, injected i.p. with FG-4592 for 2 weeks (in total 7 injections). **g** Longitudinal and transversal views of representative tibia µCT reconstructions from mice treated with FG-4592 (*n* = 6) or vehicle (*n* = 3), depicting the volume of interest (VOI) used for trabecular bone (TB) and cortical (CT) analyses. **h** µCT analysis of trabecular bone volume over tissue volume (BV/TV), trabecular thickness (Tb.Th), number (Tb.N), and separation (Tb.Sp). **i** µCT analysis showing cortical thickness (C.Th) and periosteal bone perimeter (B. Pm) in the mid-diaphysis. **j** EMCN staining (green) on tibia sections showing representative single optical slices acquired by confocal imaging (*n* = 3 vehicle, *n* = 6 FG-4592). Blood vessels were quantified in 2D in a region of interest (ROI) of 800 µm^2^ (dashed box in upper images, magnified below), including for local thickness, represented by the color gradient in the bottom images. Scale bar, 200 µm. **k** Blood vessel area, as a percentage of the ROI area (800 µm^2^). **l** Frequency distribution of blood vessel diameter obtained by local thickness quantifications. **m** Body weights monitored after 2 weeks of treatment (*n* = 4 vehicle, *n* = 8 FG-4592). **n** Glucose tolerance tests (GTT) conducted 12 h after food withdrawal, showing no significant differences in the area under the curve (AUC) between the groups (*n* = 4 vehicle, *n* = 8 FG-4592). Statistical analyses were done by one-way ANOVA (**b**, **c**) and *t*-test (**h**, **i**, **k**, **m**), with differences indicated as *P < 0.05, **P < 0.01, ***P < 0.001, ****P < 0.000 1; ns not significant

In this study, we focused on the impact of treatment with the HIF-PHD-inhibitor (PHDi) FG-4592, a small heterocyclic molecule also known as Roxadustat, on bone and energy metabolism in mice. First, to validate the capacity of the compound to activate the hypoxia signaling pathway in osteoblast lineage cells, we isolated primary osteoblasts (pOBs) from mouse calvaria and treated them with FG-4592 versus vehicle. Western blot confirmed effective HIF-1α protein stabilization after 2 h exposure to FG-4592 (Fig. 1a), associated with significant upregulation of the mRNA expression of various HIF target genes after 24 h (Fig. 1b). Parallel wells of the cells were cultured in 1% O_2_ (hypoxia) as a control for the impact of HIF-signaling activation. Compared with vehicle-exposed cells, the FG-4592-treated cells showed upregulated mRNA expression of *Vegf*, lactate dehydrogenase (*Ldha*), pyruvate dehydrogenase kinase 1 (*Pdk1*), phosphoglycerate kinase 1 (*Pgk1*) and glucose transporter 1 (*Glut1*), to a similar degree as the cells exposed to hypoxia (Fig. 1b), indicating activation of HIF’s transcriptional activity and suggesting an increase in glycolytic metabolism. Accordingly, pOBs cultured with FG-4592 or in 1% O_2_ showed increased glucose consumption (Fig. 1c). These results verify the effective activation of the hypoxia signaling pathway by FG-4592 in osteoblast lineage cells in vitro. Second, we tested the in vivo applicability of the compound by systemically administering FG-4592 to mice by intraperitoneal (i.p.) injection and assessing HIF-1α levels in different tissues a few hours later. The compound was effective in stabilizing HIF-1α in vivo, including in bone and liver, as shown by Western blot (Fig. 1d, e).

Next, to address the compound’s activity and the short-term effects of systemic HIF activation on bone health, we treated young adult CD1 mice with FG-4592 (20 mg/kg) via i.p. injections three times per week for 2 weeks (Fig. 1f). To validate the effectiveness of the drug in activating HIF-driven gene expression within bone and soft tissues, we performed quantitative reverse-transcription PCR (qRT-PCR) on bone and liver samples collected from a subset of mice at 6, 12, or 24 h after the last FG-4592 injection. The data showed upregulated expression of several HIF target genes, including *Glut1* and *Pdk1* in bone, and erythropoietin (*Epo*) and *Egln1* (the gene encoding PHD2) in liver samples, suggesting locally increased HIF activity in multiple tissues including bone (Fig. S1).

As measured by micro-computed tomography (µCT), the FG-4592 treatment significantly increased trabecular bone mass by increasing the number of trabeculae while safeguarding normal trabecular thickness (Fig. 1g, h), without affecting the cortical thickness or the periosteal bone perimeter (Fig. 1i). Moreover, FG-4592 administration increased bone vascularization, as observed by immunohistochemical (IHC) staining for the endothelial cell marker endomucin (EMCN) (Fig. 1j) and quantifications based thereon. The data show that the compound augmented the vascularized area (as fraction of tissue area) (Fig. 1k) and the frequency and size distribution of skeletal blood vessels (Fig. 1l). Of note, administration of FG-4592 in these basal conditions tended to slightly decrease the body weight of the mice over the 2-week period (Fig. 1m, *P* = 0.059 1), without major impact on global glucose metabolism as shown by their normal performance in glucose tolerance tests (GTT) (Fig. 1n).

Overall, these data show that within just 2 weeks, systemic administration of FG-4592 increased bone mass and promoted skeletal vascularization in healthy young adult mice, along with possibly inducing a reduction in body weight.

### Systemic activation of the hypoxia signaling pathway protects mice against high-fat diet (HFD)-induced obesity and glucose intolerance

The improved bone mass and vascularization and the suggestion of reduced body weight in the short-term experiment in healthy mice described above led us to speculate that pharmacological HIF activation might possibly deliver beneficial effects in the clinically relevant setting of obesity and prediabetes. Therefore, we initiated long-term experiments focused on the challenged condition of diet-induced obesity, testing the systemic effect of FG-4592 in healthy mice fed a normal diet (ND) in comparison with HFD-fed mice. For this purpose, we switched to C57BL/6J mice because this strain exhibits high sensitivity to metabolic stress compared with other strains and represents one of the best-studied models of diet-induced obesity and metabolic dysregulation, typically reflecting a prediabetic state with body weight gain, fat accumulation, and glucose intolerance, albeit without developing bona fide T2DM.^59,60^ Furthermore, we used male C57BL/6 mice because of their stronger susceptibility to HFD-induced obesity and metabolic dysfunction than female mice, with documented adverse effects on cancellous bone.^59,61,62^

**Fig. 2:**
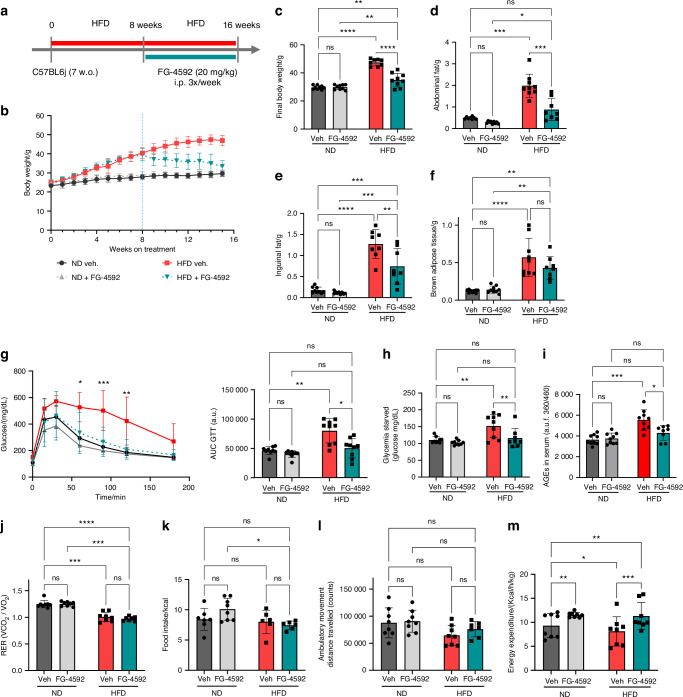
Pharmacological HIF-PHD inhibition increases energy expenditure and prevents diet-induced obesity, glucose intolerance, and peripheral fat accumulation. **a** Treatment scheme. Seven-week-old C57BL6 mice were fed a high-fat diet (HFD) or a normal diet (ND) for 16 weeks. The mice received FG-4592 (20 mg/kg) or vehicle solution via i.p. injection 3 times per week from the 8th week on diet onwards, for a total of 8 weeks (*n* = 9 mice/group). **b** Weekly follow-up of body weight. The vertical dashed line indicates the start of the treatment. **c** Final body weight after 16 weeks of ND versus HFD for groups receiving vehicle versus FG-4592 (23-week-old mice). **d**–**f** Abdominal (**d**), inguinal (**e**), and brown (**f**) fat weight, respectively, at the end of the experiment. **g** GTT after 12 h of food withdrawal, with AUC quantification. **h** Glycemia measured after 12 h fasting. **i** Circulating AGEs quantification. **j**–**m** Indirect calorimetric analysis from metabolic cages measurements during night activity (12-h monitoring period), showing respiratory exchange ratio (RER) (**j**); food intake (kcal) (**k**); ambulatory movement (**l**); and energy expenditure (**m**) (*n* = 8 mice/group). Statistical analyses were done by two-way ANOVA. There was a statistically significant interaction between the effects of diet and treatment on parameters related to obesity (**c**–**e**) and glucose homeostasis (**g**–**i**), but not on brown fat (**f**) and indirect calorimetry parameters (**j**–**m**). Multiple comparisons results among all groups are shown as *P < 0.05; **P < 0.01; ***P < 0.001; ****P < 0.000 1; ns not significant

As schematically depicted in Fig. 2a, the HFD was started at 7 weeks of age and continued for a total of 16 weeks, with FG-4592 treatment or vehicle administration starting from the 8th week on the diet onwards (20 mg/kg i.p., 3 times per week) (Fig. 2a). Animals fed a ND showed minor age-related body weight increases (Fig. 2b), without impact of FG-4592 administration (Fig. 2b, c). As expected, HFD induced significant body weight gain, and this increase continued in the mice receiving vehicle injections over the 8-week period of the treatment. In sharp contrast, PHD inhibition by FG-4592 administration immediately blocked further body weight increases in mice receiving HFD, and even reverted the body weight almost back to ND levels by week 16 (Fig. 2b, c). In line with this protection against weight gain in HFD-fed animals, FG-4592 effectively reduced peripheral fat accumulation, thereby fully preventing the HFD-induced increase in abdominal white adipose tissue (WAT) mass and significantly reducing the expansion of the inguinal fat (Fig. 2d, e). The interscapular brown adipose tissue mass also increased in mice on HFD compared with those on ND, yet this effect was not altered by PHDi treatment (Fig. 2f). PHD inhibition also completely recovered the mice from diet-induced glucose intolerance (Fig. 2g) and prevented the HFD-associated high glycemia levels seen in vehicle-treated animals (Fig. 2h). Concomitantly, the increased AGEs levels in the serum of HFD-fed mice were completely normalized by FG-4592 treatment (Fig. 2i). Together, these data show that PHD inhibition using FG-4592 can effectively protect mice from the adverse metabolic consequences of a long-term fatty diet, alleviating the obesity, glucose intolerance, and excessive circulating AGEs. These findings highlight the beneficial metabolic impact of systemic HIF activation in a mouse model of obesity.

### Roxadustat administration increases energy expenditure in vivo

We next performed indirect calorimetric analysis to evaluate the mechanism underlying these major metabolic changes observed upon HFD and PHDi administration. As expected, HFD-fed animals showed a decreased respiratory exchange ratio (RER) of CO_2_ production over O_2_ consumption (VCO_2_/VO_2_) compared to animals on ND, reflecting the shift towards higher nutrient usage of fat through fatty acid oxidation; this parameter was independent of whether the mice received vehicle or FG-4592 (Fig. 2j). Neither the diet nor the treatment affected the food intake (in kcal) (Fig. 2k) or the ambulatory movement (Fig. 2l). Interestingly, however, the metabolic cage recordings revealed a significant increase in energy expenditure (or heat production) in the groups treated with FG-4592 (Fig. 2m), which could explain its beneficial impact on global metabolism.

### Systemic hypoxia signaling pathway activation prevents HFD-induced medullary fat accumulation and vascular damage in the skeleton

**Fig. 3:**
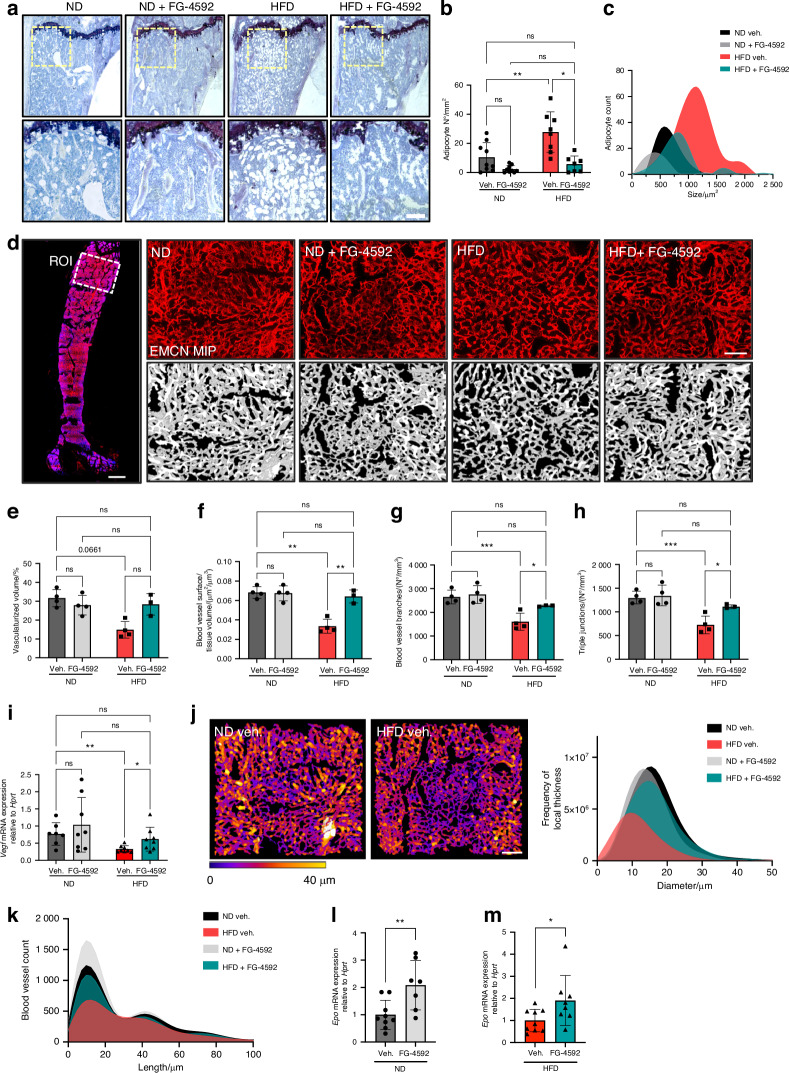
Pharmacological activation of the hypoxia signaling pathway prevents HFD-induced medullary fat accumulation and vascular damage in the skeleton. **a** BM adipose tissue (BMAT) visualization by toluidine blue staining in tibia sections; lower panels: magnified views of the metaphyseal region below the growth plate. Scale bar, 100 µm. **b** Adipocyte numbers, as quantified in the metaphysis (*n* = 7–9 mice/group). **c** Frequency distribution of adipocyte size measured by area per cell in 2D. **d** Left panel: single optical image showing a full femur section stained for EMCN (blood vessels, red) and Hoechst (nuclei, blue) depicting the metaphyseal ROI, comprising 1.5 × 1.5 mm, placed under the growth plate; scale bar, 500 µm. Right panels, upper row: maximum intensity projection (MIP) of confocal images of EMCN-stained blood vessels (100 µm depth); scale bar, 200 µm. Lower row: binarized 3D vascular network (white) obtained from semi-automated segmentation of EMCN staining. **e**–**h** Morphological analyses of the skeletal vasculature, showing vascularized volume relative to the ROI tissue volume (µm^3^/µm^3^ expressed in %) (**e**); blood vessel surface over tissue volume (µm^2^/µm^3^) (**f**); blood vessel branches (number/µm^3^ tissue volume), calculated as single strings from node to node in a skeletonized network (**g**); triple junctions of the vascular network depicting web complexity, calculated as nodes with 3 branches (**h**). **i** qRT-PCR of *Vegf* mRNA expression levels in full humerus extracts, normalized to *Hprt*. **j** Analysis of blood vessel diameters, showing (left) representative images of gradient pseudo-coloration from blue to yellow (0–40 µm thick) (scale bar 200 µm) and (right) the frequency distribution of local thickness. **k** Frequency distribution for the blood vessel branch lengths, considered as lengths of each single string from node to node. **l** qRT-PCR of *Epo* levels relative to *Hprt* expression in humeri harvested from healthy mice. **m** *Epo* mRNA levels relative to *Hprt* expression in bones from vehicle-injected versus FG-4592-treated HFD-fed mice. Statistical analyses were done by two-way ANOVA, with multiple comparisons between diets and treatments; *n* = 8 (adiposity analyses); *n* = 3–4 (vascular analyses); *n* = 7–9 (qRT-PCR analyses). *P < 0.05; **P < 0.01; ***P < 0.001; ****P < 0.000 1; ns not significant

Given the reduced peripheral fat accumulation in mice treated with FG-4592, and the fact that HFD is also associated with excessive medullary fat accumulation,^26,63^ we analyzed whether hypoxia signaling activation impacted BM adiposity. On histological bone sections, we observed a prominent accumulation of adipocytes in the proximal region of the tibia in vehicle-treated HFD-fed animals, as confirmed by counting the cells in a standardized metaphyseal region (Fig. 3a, b). Morphometric analysis at the cellular level denoted a higher frequency of large adipocytes in the HFD-fed group (Fig. 3c). Remarkably, FG-4592 treatment fully protected animals against this HFD-induced BMAT accumulation and hypertrophy (Fig. 3a–c). In fact, even on ND, FG-4592 tended to reduce the amount and size of BM adipocytes (Fig. 3a–c).

Next, we evaluated the effects on bone vascularization by staining for EMCN and performing confocal 3D-imaging in the femoral metaphyseal region, followed by quantitative image analysis of the vascular network (Fig. 3d–h). We observed a marked deterioration of the skeletal vasculature in HFD-fed animals (receiving vehicle), displaying a decreased vascular volume, reduced blood vessel surface, and reduced numbers of vascular branches and junctions compared with the healthy, ND-fed mice (Fig. 3e–h). These morphological features overall uncover a detrimental impact of HFD on the bone vascular network, both in terms of density and complexity. Notably, FG-4592 administration completely prevented the HFD-associated skeletal vascular decline, recovering the vascular surface density and network complexity, as represented by normal numbers of vessel branches and junctions (Fig. 3d–h). In line with this protection against microvascular degeneration in bone, PHDi administration fully counteracted the reduced *Vegf* expression observed in bones from HFD-fed animals (Fig. 3i). Deepening the analysis of the vascular changes, we further calculated the local thickness of blood vessels segmented in 3D (Fig. 3j), finding the frequency distribution of the local thickness to indicate a decrease in the prevalence of small blood vessels as well as in the presence of blood vessels with larger diameters in HFD-fed animals (receiving vehicle). PHDi treatment recovered the number and diameter of the blood vessels in HFD-exposed mice (Fig. 3j). We also calculated blood vessel length, as the distance of each single string running from node to node in the skeletonized vascular network, uncovering a decreased frequency of short blood vessels (<20 µm) in HFD-fed animals, which was restored in FG-4592-treated HFD-fed animals, to the levels seen in untreated ND-fed animals (Fig. 3k).

Of note, the vascular decline in the BM of HFD-fed animals was not associated with functional impairments in hematopoiesis, as judged by the normal levels of circulating blood cell types (Fig. S2). FG-4592 administration by itself did not impact blood cell frequencies over the shorter 2-week treatment period (although platelets seemed somewhat sensitive) (Fig. S2a), but led to increased red blood cells, hematocrit, and hemoglobin levels in the blood after 8 weeks of treatment in the context of HFD (Fig. S2b). This side-effect is in line with the strong induction of *Epo* expression by HIF, which is observed even in bone tissue (where *Epo* is normally hardly or not expressed) upon genetic *Vhl* inactivation^64^ or upon PHDi treatment as seen in the current study (Fig. 3l, m). The induction of EPO production is also the basis of the use of Roxadustat and other PHD inhibitors as therapeutics for anemia in chronic kidney disease (CKD).^34,35,65^

Together, these analyses of the bone microenvironment revealed that pharmacological HIF activation by FG-4592 effectively counteracts the accumulation of BM adipocytes and prevents the decline in the skeletal vascular network in the context of diet-induced obesity and prediabetic metabolic dysfunction.

### Activation of the hypoxia signaling pathway promotes bone formation

**Fig. 4:**
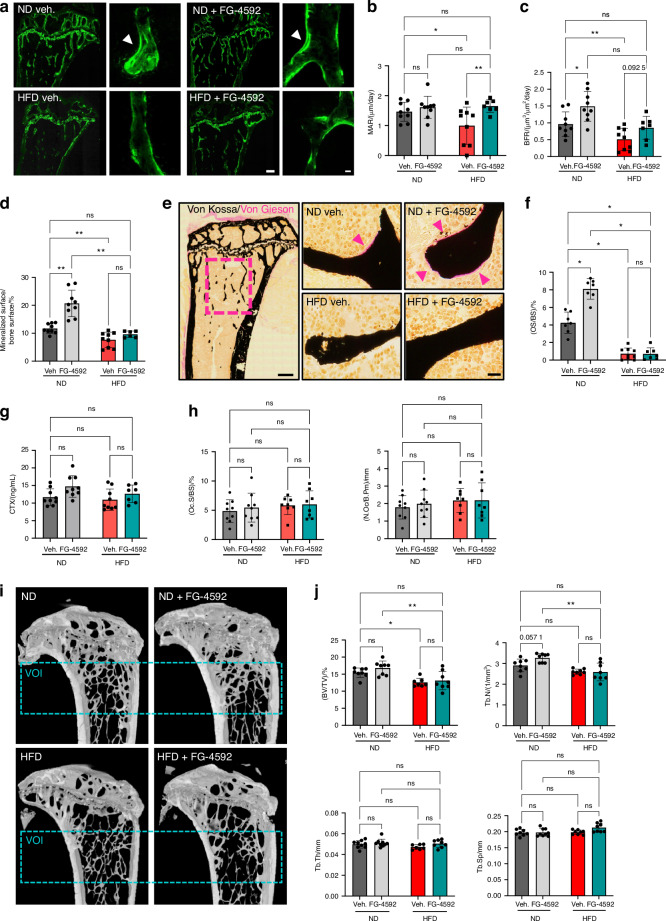
Activation of the hypoxia signaling pathway promotes bone formation without causing major changes in bone mass in diet-induced metabolic stress conditions. **a**–**d** Calcein label-based dynamic histomorphometry, showing representative images (**a**), mineral apposition rate (MAR) (**b**), bone formation rate (BFR) normalized to bone surface (**c**), and mineralized surface (as % of bone surface) (**d**). **e** Representative images of Von Kossa/Von Gieson staining on undecalcified tibia MMA sections, showing mineralized bone (black) and non-mineralized osteoid (pink, pointed by arrowheads); scale bar, 500 µm in overview and 50 µm in magnifications. **f** Osteoid surface normalized to bone surface (OS/BS, in %) measured in the metaphyseal bone region as boxed in (**e**). **g** CTX levels in the serum of the mice. **h** Histomorphometric analysis of TRAP-positive cells visualized in tibia sections, showing no significant differences between the groups in the osteoclast surface [relative to bone surface (Oc.S/BS)] and osteoclast number (N.Oc) relative to bone perimeter (B.Pm). **i** Longitudinal µCT views of tibias from mice on ND or HFD and treated with FG-4592 or vehicle, depicting the VOI used for trabecular bone analysis. **j** µCT analysis, depicting trabecular bone volume over tissue volume (BV/TV), trabecular number (Tb.N), thickness (Tb.Th), and separation (Tb.Sp). Statistical analyses were done by two-way ANOVA, with multiple comparisons between diets and treatments; *P < 0.05; **P < 0.01; ***P < 0.001; ****P < 0.000 1; ns not significant. Significant interaction between diet and treatment was seen for the bone formation parameters in (**c**–**f**) but not for µCT data (**j**). All analyses were done on *n* = 7–9 mice/group

Given the impact on trabecular bone mass that we observed already after short-term FG-4592 treatment in healthy mice (see Fig. 1g, h), we next assessed the bone phenotype of the mice exposed to the longer-term PHDi treatment in the HFD context (Fig. 4). First, to evaluate the process of mineralized bone formation, we performed dynamic bone histomorphometry by calcein double labeling. The results indicate that HFD induced a considerable and significant decrease in the mineral apposition rate (MAR), the bone formation rate (BFR), and the mineralized surface in animals on a HFD regimen (Fig. 4a–d). Remarkably, the MAR was fully recovered to normal levels in the PHDi-treated group on HFD (Fig. 4b), and the BFR tended to be less severely suppressed as well (Fig. 4c). In ND, FG-4592 administration significantly increased the BFR and mineralized surfaces (Fig. 4c, d). The reduced mineralized bone formation in HFD-fed animals was associated with drastically suppressed deposition of osteoid (unmineralized bone matrix), as shown by histomorphometric analysis on Von Kossa/Van Gieson-stained sections (Fig. 4e, f). Although the virtual absence of osteoid observed in HFD conditions was not recovered by PHDi treatment, FG-4592 supplementation did significantly increase the osteoid levels in ND-fed animals (Fig. 4e, f). In contrast to these evident alterations observed in bone formation parameters, bone resorption did not appear to show notable changes by either the diet or the treatment in our experiment. The circulating CTX levels, a measure of collagen degradation products denoting overall bone resorption, were similar in each of the four groups (Fig. 4g). Moreover, histomorphometric analysis of TRAP-stained cells on the trabecular bone surfaces revealed no significant differences in the osteoclast surface (relative to bone surface) and osteoclast number between any of the groups (Fig. 4h).

To evaluate the net impact on trabecular bone mass, we analyzed the tibias by µCT (Fig. 4i, j). Mice on the HFD regimen showed a reduced trabecular bone volume over tissue volume (BV/TV), in line with the reduced bone formation in this setting (see Fig. 4a–f), although we did not observe major changes in trabecular number, thickness, or separation in our cohort (Fig. 4j). In ND conditions, we found a tendency to an increased trabecular number and slightly higher BV/TV in FG-4592-treated animals compared with those receiving vehicle, in line with the observations made after 2-week treatment (see Fig. 1h). However, under the stress of a 16-week HFD, the partial (8-week) PHDi administration did not significantly affect the final trabecular bone parameters (Fig. 4j).

Thus, we found that FG-4592 treatment stimulates bone formation and counteracts the inhibitory impact of HFD on bone mineralization, but overall, these effects cumulated in merely mild changes in bone mass in the trabecular bone compartments of the tibia within this period of treatment.

### Activation of the hypoxia signaling pathway does not cause major changes in cortical bone mass, intracortical vasculature, and bone matrix composition

**Fig. 5:**
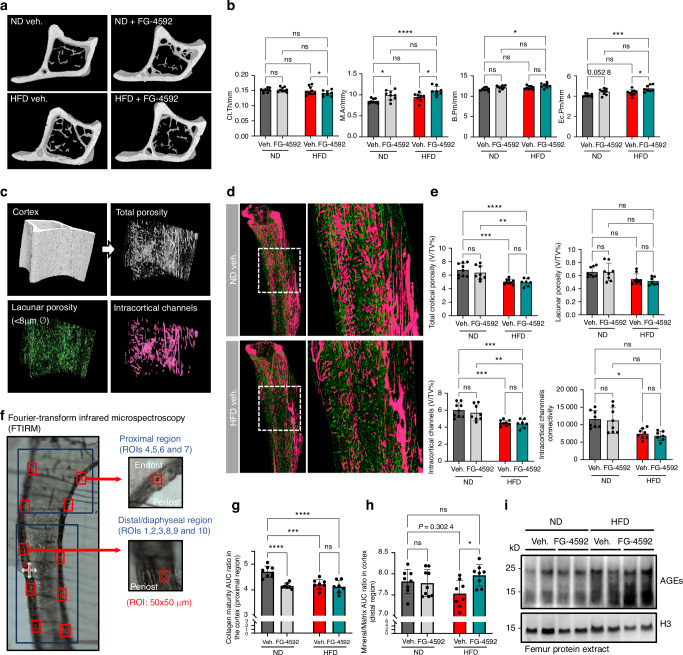
Hypoxia signaling pathway stimulation does not cause major changes in cortical bone mass, porosity, and bone matrix composition. **a** Transversal µCT views of the mid-diaphysis of tibias from mice on ND or HFD and treated with FG-4592 or vehicle. **b** Cortical bone µCT analysis, showing cortical thickness (C.Th), medullary area (M.Ar), periosteal bone perimeter (B.Pm), and endocortical bone perimeter (Ec.Pm) measured 2.5 mm below the growth plate. **c** Illustration of the cortical porosity segmentation pipeline. µCT scans at 5 µm voxel size were segmented into mineralized and non-mineralized cortex; non-mineralized intracortical space was then segmented into lacunar porosity (<8 µm Ø) and intracortical channels (>8 µm Ø). **d** Representative images of cortical porosity of vehicle-injected animals on ND versus HFD, showing lacunar porosity in green and intracortical channels in pink. **e** Quantification of total cortical porosity, lacunar porosity, and intracortical channels (all in volume % relative to cortical volume), and of the intracortical channels’ connectivity, depicting network complexity. **f** FTIRM analysis outline, showing a representative tibia MMA section depicting the localization of the different ROIs in the proximal (ROI 4–7) and distal/diaphyseal (ROI 1–3, 8–10) regions of the cortex (red squares, 50 × 50 µm each). **g**-**h** FTIRM results, showing collagen maturity in the proximal cortex (**g**), and mineral to matrix ratio in the diaphyseal cortex (**h**). For details, see Fig. S3. **i** Western blot for AGEs in femur protein extracts, with histone H3 as loading control (*n* = 2/group). Statistical analyses were done by two-way ANOVA, with multiple comparisons between diets and treatments; *n* = 7–9 mice/group; *P < 0.05; **P < 0.01; ***P < 0.00 1; ****P < 0.000 1; ns, not significant. Interaction between the diet and treatment was statistically significant for the data shown in (**b**) (except for Ct.Th.) and (**g**)

We also performed an in-depth analysis of the cortical bone of the mice included in the HFD experiment, using several high-resolution techniques. First, imaging of the tibias by µCT revealed that, in general, the cortical bone thickness was hardly affected by the diet or the PHDi regimen (Fig. 5a, b). No significant HFD-induced changes were seen in the geometry of the cortical bone shaft, as measured by the medullary area (M.Ar), outer bone perimeter (B.Pm), and inner endocortical perimeter (Ec.Pm) (Fig. 5b). Notably, a slight but statistically significant widening of the marrow cavity at the level of the mid-diaphyseal bone shaft was observed in mice treated with FG-4592, in both the ND and HFD conditions, which seemed to be explained mostly by changes at the endosteal side rather than by periosteal accrual (Fig. 5b). Altogether though, within the period of the treatment and the diet we documented merely mild changes in the geometry of the cortical bone.

To further assess the microstructure of the cortical bone, we evaluated the cortical porosity using the µCT scans of the tibias. Besides calculating the total porosity, we classified the pores into lacunar porosity (isolated structures with a diameter below 8 µm) and intracortical channels (>8 µm Ø), the latter of which host in part the cortical blood vessels^66,67^ (Fig. 5c, d). Intriguingly, these intracortical channels and their connectivity were profoundly reduced in the HFD-fed animals in our cohort, cumulating in a decreased global cortical porosity despite minor (non-significant) impact on the lacunar porosity (Fig. 5e). However, neither the intracortical channel volume, nor the channels’ connectivity was affected by FG-4592 administration in ND or HFD conditions (Fig. 5e). Overall, these data indicate that PHDi treatment does not affect the cortical pores and channels, and does not protect against HFD-induced changes in cortical porosity in the mice and context of our study.

Second, to analyze the matrix composition and microstructural organization of the cortical bone, we performed Fourier-transform infrared microspectroscopy (FTIRM) on MMA-embedded tibias, analyzing proximal regions of the cortex (adjacent to the metaphysis) as well as regions located deeper into the diaphyseal shaft (Fig. 5f). Collagen maturity in the proximal cortex was found to be reduced in the HFD-fed group compared to animals on ND (Fig. 5g). FG-4592 reduced collagen maturity in ND conditions but did not further impact collagen maturation in the HFD regimen (Fig. 5g). The mineral-to-matrix ratio in the diaphyseal cortex tended to be lower in HFD-fed animals, and rescued by FG-4592 (Fig. 5h). Other parameters, including carbonation, mineral maturity and crystallinity, showed no significant differences between the groups in either of the cortical regions analyzed (Fig. S3). Thus, overall, only mild changes were observed in the cortical bone composition and structural organization upon HFD and/or FG-4592 treatment in the mice studied here.

Altogether, the data presented here highlight the adverse impact on the skeleton of excessive dietary fat intake, obesity, and glucose intolerance, with major changes occurring in the BM microenvironment and trabecular bone formation, which are rescued by PHDi administration. Notably, despite the profound improvement of global glucose handling by FG-4592, the HFD-induced increased accumulation of AGEs locally in bone was not affected by the treatment (Fig. 5i). This may suggest that the protective effects of HIF activation on BM adiposity and skeletal vascularization were independent from the local deposition of AGEs.

### PHD inhibition improves systemic glucose metabolism and prediabetic fracture repair

Given the FG-4592-triggered metabolic benefits and improved bone formation and vascularization observed in mice with diet-induced obesity, we next sought to evaluate the capacity of the PHDi treatment to support glucose handling and bone regeneration in a model of metabolically compromised fracture repair. We opted for a non-stabilized tibia fracture model, as employed by others before to study delayed or nonunion healing,^68–70^ to reflect a compromised fracture repair situation, thus providing a fitting model for testing the potential pro-regenerative effect of the PHDi treatment in obese/prediabetic conditions.

**Fig. 6:**
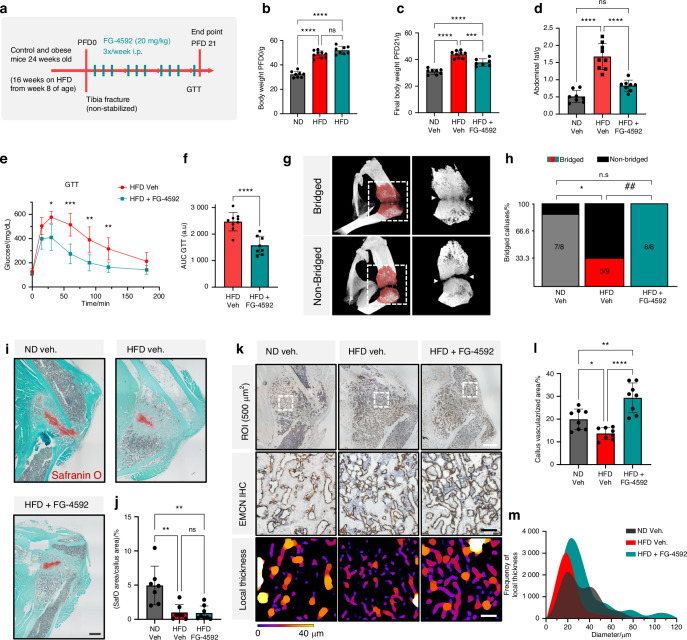
Administration of FG-4592 during fracture healing in obese/prediabetic mice improves both global glucose metabolism and bone regeneration. **a** Experimental setup. C57BL6 mice were on a HFD or ND regimen for 16 weeks, from 8 to 24 weeks of age, and subsequently subjected to a non-stabilized tibia fracture. FG-4592 (20 mg/kg i.p.) or vehicle was given 3 times per week from post-fracture day (PFD) 2 until PFD 21. **b**-**c** Body weight on the day of fracture (PFD 0) (**b**) and at PFD 21 (**c**). **d** Abdominal fat weight at the end of treatment (PFD 21). **e**-**f** GTT at PFD 20 (**e**), with AUC quantification (**f**). **g** Representative µCT reconstructions illustrating bridged versus non-bridged tibia fractures; newly formed callus tissue is pseudo-colored in red, and white arrows point at the mineralized (top) and non-mineralized (bottom) fracture sites. **h** Proportion of bridged vs non-bridged calluses in each condition. Callus bridging proportions were analyzed by exact Fisher’s test of categorical data. See Fig. S5, for views on all the individual calluses. **i** Representative images of non-stabilized tibia fractures stained with Safranin O depicting the cartilage in the callus (red). Scale bar, 500 µm. **j** Quantified Safranin O-stained area, expressed as % of the callus area [µm^2^/µm^2^ (%)]. **k** EMCN IHC showing the callus vasculature in each condition; in a 500 × 500 µm ROI (dashed box in upper images, magnified below) the blood vessels were segmented and quantified, including for local thickness (represented by the color gradient in the bottom images). Scale bar, 100 µm. **l** Vascularized area as % of the callus ROI. **m** Frequency distribution of blood vessels’ diameter (μm) obtained from local thickness quantifications. Statistical analyses were done by one-way ANOVA (**b**–**d**, **j**, **l**), *t*-test (**e**, **f**), or Fisher’s exact test of categorical data (**h**) (with *, comparison between ND and HFD vehicle groups; #, comparison between HFD-vehicle and HFD-FG-4592-treated groups). Significant differences are indicated as *P < 0.05; **P < 0.01; ##, *P* < 0.01; ***P < 0.001; ****P < 0.000 1; ns non-significant

We fractured 24-week-old mice that had been on a HFD (versus ND controls) for 16 weeks prior to the trauma induction, and we administered FG-4592 (20 mg/kg i.p.) or vehicle solution 3 times per week during the first 3 weeks of the repair process. A final injection with FG-4592 was given at the endpoint, post-fracture day 21 (PFD 21) (Fig. 6a), and the fracture callus, soft tissues, and contralateral uninjured bones were collected 1 h afterwards. Gene expression analysis in femurs from the non-injured contralateral leg revealed that the PHDi treatment led to upregulated expression of *Glut1* in bone (but not of other HIF target genes), whereas liver samples showed increased expression of multiple HIF-responsive genes at this stage (including *Epo*, *Vegf*, *Phd2*, *Glut1*, and *Ldha*, but not *Pgk1* and *Pdk1*) (Fig. S4), confirming the efficacy of the systemic treatment.

A baseline assessment (at PFD 0) confirmed that the mice that had been fed a HFD for 16 weeks showed substantially higher body weights than chow-fed mice, i.e., prior to the fracture induction and the start of PHDi treatment (Fig. 6b). Remarkably, by PFD 21, HFD-fed mice that received FG-4592 during the 3-week regeneration process had lost weight, and now showed a markedly reduced body weight in comparison to vehicle-injected HFD-fed mice (Fig. 6c). The decrease in body weight aligned with a reversal of the abdominal fat accumulation, that completely normalized to ND levels (Fig. 6d). Moreover, PHDi administration over the 3-week period led to significant improvement of the GTT (Fig. 6e, f).

Analyzing the progress of bone regeneration, we categorized fractures into “bridged” versus “non-bridged” by 3D µCT analysis of the PFD 21 calluses. Bridged calluses displayed a clear presence of mineralized tissue at the level of the fracture line (evaluated in 3D), connecting the proximal and distal parts of the broken tibia, whereas non-bridged calluses showed a non-mineralized fracture line (Fig. 6g). While bridging was observed in nearly all mice in ND conditions (in 7 out of 8 animals, i.e., *n* = 7/8), HFD-fed animals displayed a markedly reduced regenerative capacity, dropping to around 30% of bridged events (*n* = 3/9, Fig. 6h). Remarkably, HFD-fed animals treated with FG-4592 showed a full recovery of the bridging capacity, with 100% of the calluses showing bridging (*n* = 8/8, Fig. 6h and Fig. S5 showing all the individual mouse calluses). To understand the mechanisms by which FG-4592 improved the compromised fracture regeneration, we analyzed histological sections for the presence of cartilage (by Safranin O staining) and blood vessels (by EMCN IHC). In line with other reports,^29^ we observed a drastically decreased proportion of cartilage in the calluses of HFD-fed mice at PFD 21 (Fig. 6i, j), but this reduction in callus cartilage was not affected by FG-4592 (Fig. 6i, j). On the other hand, impaired callus vascularization in HFD-fed animals, revealed by EMCN IHC analysis, was effectively recovered by the PHDi treatment (Fig. 6k–m). The HFD-associated decrease in the blood vessel area in the callus was countered by FG-4592, to levels that even exceeded those in ND-fed mice (Fig. 6l). Frequency distribution of blood vessel size showed an increased number of smaller vessels (<500 µm^2^, equivalent to a ~25 µm diameter), but almost absent large blood vessels (>50 µm diameter) in HFD conditions with vehicle administration (Fig. 6m). PHDi treatment recovered the frequency of the large blood vessels, shifting the distribution curve of vessel diameter towards the ND signature (Fig. 6m).

Altogether, these data show that systemic administration of the PHD inhibitor FG-4592 during fracture repair in metabolically challenged conditions improves bone tissue regeneration, at least in part by promoting angiogenesis in the callus and stimulating tissue revascularization, along with ameliorating HFD-induced obesity and glucose intolerance.

## Discussion

**Fig. 7:**
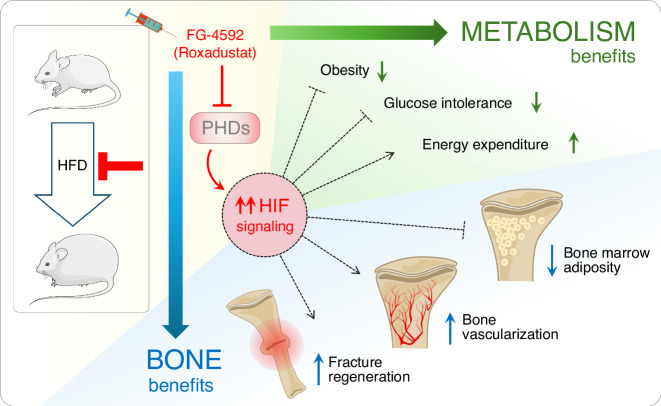
Scheme summarizing the findings presented in this manuscript. Obesity commonly leads to devastating effects on many organs, including bone, and can be the prelude to developing hyperglycemia and diabetes, typically reducing bone quality, increasing fracture risk, and impairing bone regeneration. The key roles of hypoxia-inducible factor (HIF) signaling in regulating cellular metabolism and glucose uptake, angiogenesis, and bone biology and repair, suggested that HIF activation might concomitantly improve systemic glucose homeostasis and bone health and regenerative potential in the context of obesity and metabolic stress. Here, we tested this hypothesis by systemically administrating the HIF-prolyl-hydroxylase domain (PHD) inhibitor FG-4592 (Roxadustat) in mice challenged with a high-fat diet (HFD). Our findings reveal that pharmacological activation of the hypoxia signaling pathway can effectively reduce diet-induced body weight gain and peripheral fat accumulation as well as glucose intolerance, by increasing global energy expenditure. Concomitantly, the activation of HIF signaling was shown to improve bone health in obesogenic conditions by blocking excessive adipocyte accumulation in the bone marrow and safeguarding the bone vascular system, as well as enhancing metabolically compromised fracture repair. Thus, therapeutic HIF activation concomitantly alleviates both the metabolic and skeletal consequences of obesity and prediabetes in mice

The metabolic disorders obesity and diabetes constitute a global epidemic, disrupting glucose homeostasis and adversely affecting multiple organs, including bone. The association of these conditions with reduced bone quality, increased fracture incidence, and impaired fracture healing is a growing clinical concern, particularly given the current lack of targeted therapeutic solutions.^6–11,27,31,32^ In fact, many lifestyle and surgical interventions promoting body weight loss, which is the cornerstone in the management of obesity with or without T2DM, and some classes of glucose-lowering diabetes medications, are associated with an adverse impact on bone, increasing the risk of bone loss and fractures. Thus, there is a large need for research into metabolism-improving treatment strategies that are protective, rather than deleterious, for bone.^6,7,27^ Here, we evaluated the potential therapeutic benefits of HIF-PHD inhibitor administration to improve bone homeostasis and repair in the context of diet-induced obesity and metabolic dysfunction in mice, based on the premise that pharmacological activation of the hypoxia signaling pathway could possibly constitute a double-targeting approach, alleviating both the systemic metabolic impact and the local complications in the skeleton by acting in various tissues as well as locally promoting glucose utilization and cellular glycolysis, bone formation, and angiogenesis, similar to genetic VHL-inactivation in osteoblasts in our preceding studies.^49,57^ Our results demonstrate that systemic administration of FG-4592 (Roxadustat) effectively mitigated HFD-induced obesity and prediabetes symptoms in mice, protecting the animals against body weight gain, glucose intolerance, and peripheral fat accumulation (Fig. 7). Mechanistically, indirect calorimetry revealed that the broad-acting HIF-activating treatment systemically increased energy expenditure. Concomitantly, bone health and regeneration were markedly improved in the context of the obesogenic diet, plausibly due to the metabolic improvement as well as direct HIF effects in bone cells (Fig. 7). The latter is supported by the finding that short-term administration of FG-4592 in healthy conditions led to markedly increased trabecular bone mass, hence showing the potential of the drug to impact the skeleton independent from its metabolic impact. In the HFD model employed here, FG-4592 administration completely recovered the skeletal vascular damage, the excessive expansion of the BM adiposity, and the bone mineral apposition deficits that were induced by the diet. Moreover, providing FG-4592 during the regeneration of a non-stabilized bone defect in this metabolically challenged context improved the fracture repair capacity, again by activating HIF-responses in soft tissues and bone and promoting global glucose homeostasis, bone formation and angiogenesis (Fig. 7). Altogether, this work suggests that pharmacological activation of the hypoxia signaling pathway through HIF-PHD inhibition might be a valuable therapeutic strategy to concomitantly alleviate the metabolic and skeletal consequences of obesity and, possibly, of T2DM. Interestingly, PHD inhibitors are already in clinical use for the treatment of anemia associated with CKD, based on their erythropoiesis-stimulating effect by enhancing the production of EPO, a direct target of HIF-2α.^34,35,65^ FG-4592/Roxadustat has been approved for this purpose and is currently in use in Europe, Japan, China, Chile, and South Korea, constituting an alternative, oral therapy that can replace the subcutaneous administration of recombinant human EPO, which is both expensive and inconvenient for the patient.^65^ In addition to Roxadustat, the patent of which expired in June 2024, several other small-molecule HIF-PHD inhibitor drugs with disclosed chemical structure have progressed to registered clinical trials and/or FDA approval for anemia in patients with CKD.^35^

The idea that small-molecule HIF stabilizers might be useful to improve glucose and lipid metabolism has been raised before, mostly based on a few preclinical research studies. For instance, a compound different from the one used in our study, FG-4497, was shown to be protective against HFD-induced obesity in mice, decreasing body weight and WAT, and to improve glucose tolerance.^58^ Another HIF-PHD-modulating compound, Enarodustat (JTX-951), lent protection against metabolic dysfunction in the diabetic ob/ob mouse model, by reducing body weight and fat tissue, insulin resistance, glycemia, and cholesterol levels.^71^ This compound also ameliorated multiple obesity-related consequences in HFD-fed mice,^72^ although bone was not investigated. Interestingly, individuals residing at higher altitudes have been shown to exhibit an inverse relationship with obesity prevalence and appear less susceptible to suffer from impaired glucose homeostasis and develop T2DM, a phenomenon that might perhaps relate to the diminished atmospheric oxygen levels and subsequent activation of hypoxia-related pathways.^73^ Mechanistically, the reasons why HIF activation has a net beneficial impact on global metabolism are complex and multifactorial, involving a combination of numerous effects in various tissues and organs. Evidence suggests that, notwithstanding the alleged occurrence of aberrant tissue hypoxia, the activation of HIF signaling is impaired in conditions of metabolic dysfunction such as diabetes, through various mechanisms.^33^ Most prominently, high glucose levels inhibit HIF-1 activity in several tissue cell types by reducing both its protein stability and functioning.^33^ For instance, hyperglycemia induces accumulation of the metabolite methylglyoxal, a toxic by-product of glycolysis and precursor of AGEs, which can promote HIF-1α degradation^74^ as well as repress its transcriptional activity by inhibiting heterodimerization with HIF-1β^75^ and recruitment of the co-activator p300.^76^ In addition, ROS overproduction in hyperglycemia^77^ promotes methylglyoxal accumulation^75^ and PHD activation,^78^ leading to HIF-1α degradation. Besides hyperglycemia, hyperlipidemia, and elevated levels of long-chain fatty acids may also contribute to HIF dysregulation, by preventing HIF-1α activity and promoting PHD-mediated HIF degradation.^79^ These findings support the idea that HIF-activating compounds could be successful through their ability to restore the compromised hypoxia signaling in metabolically challenged conditions.^33^ Moreover, stimulating HIF activity can promote vascular health through the induction of VEGF, which could add to alleviating macro- and microvascular complications that are the main causes of many obesity- and diabetes-related comorbidities, including retinopathy, nephropathy, neuropathy, and cardiovascular disorders.^30^ Altogether, these preceding findings and the work presented here support further research and clinical exploration of the usefulness of HIF-PHD inhibitors for improving metabolism, in obesity as well as diabetes, considering that in-depth, long-term investigations will be necessary to ensure their safety in clinical practice, given the substantial roles of HIF signaling in cancer.^80,81^

Although the preceding metabolic studies had not considered the potential impact on the skeleton, an envisioned use of PHD inhibitors to lower body weight and improve energy metabolism would be conceivable to come with coupled bone health benefits. Surprisingly, though, previous animal studies supporting the effectiveness of PHD inhibition for improving bone mass^52,53^ and regeneration^46,54,55^ have only been conducted in healthy conditions. Here, as we directed our focus specifically to metabolically challenged conditions by using a mouse model of diet-induced obesity, we now found that systemic FG-4592 administration could protect bone from HFD-induced damage and promote metabolically compromised fracture regeneration. Although the mechanisms by which metabolic dysfunction affects the skeletal system remain incompletely understood, active research suggests involvement of altered cellular metabolism and signaling, pro-inflammatory cues disrupting bone remodeling, impaired bone material properties, vascular decline, cellular senescence, and skewed differentiation and compromised regenerative responsiveness of SSPCs, collectively increasing fracture risk and hindering repair.^9,14–21,23,27^ In the PHDi treatment used here, based on the presented data we believe the skeletal benefits are related to a combination of the drug’s effectiveness in locally stimulating angiogenesis, suppressing BM adipogenesis, and promoting mineralized bone formation on the one hand, along with indirect contributions resulting from the improved systemic control of global glucose homeostasis, obesity, and inter-organ crosstalk involving bone on the other. These various aspects are elaborated on in the following paragraphs.

Firstly, by performing an in-depth analysis of the BM vascular network, our study revealed that HFD profoundly impacts the skeletal vasculature, leading to a reduced blood vessel density and deterioration of the vascular network architecture and complexity. These changes were associated with suppressed expression of VEGF, the prime mediator of physiological and pathological angiogenesis and critical regulator of osteo-angiogenic coupling in bone.^36,38,39,42–45^ In our mouse model, FG-4592 treatment normalized the VEGF levels in bone and fully preserved the density and quality of the BM vasculature. Moreover, administration of the PHD inhibitor also promoted callus vascularization, associated with improved fracture repair. These findings are in line with studies showing that VEGF upregulation reflects a key physiological role of HIF signaling in chondrocytes and osteolineage cells, as well as a prime outcome of forced HIF hyperactivation in bone cells.^45,47,49^ Many studies have furthermore shown that increased VEGF levels and bone hypervascularization are typically associated with increased osteoprogenitors and trabecular bone formation, along with high Wnt/β-catenin activity,^44^ likely explaining part of the osteogenic benefit of FG-4592. The vascular protection exerted by PHD inhibition could be quite unique and highly clinically relevant, as T2DM in humans is associated with alterations in the BM vasculature and pericytes,^16^ and the presence of micro- and macrovascular complications in patients with T2DM has been linked to higher fracture risk.^11,82^

Secondly, accumulation of adipocytes in the bone microenvironment and fracture callus is a typical feature of obesity and diabetes in humans and animals,^9,19–22,26,63^ that is increasingly speculated to contribute substantially to the pathophysiology of the metabolic dysfunction-associated bone complications.^9,20^ On the one hand, BMAT can locally impact negatively on bone quality and strength, favoring bone fragility and enhancing fracture risk. On the other hand, BMAT is a considerably large and metabolically active fat depot that is likely to exert reciprocal endocrine impact on systemic metabolic homeostasis.^9,83–86^ Remarkably, while the BMAT expansion with HFD was indeed very pronounced in our study, this feature was completely suppressed by the PHDi treatment. Since a rise in BM adiposity is reflective of increased adipocyte differentiation of SSPCs, commonly occurring at the expense of SSPC commitment toward osteoblastogenesis, dedicated follow-up research to understand the underlying molecular mechanisms is of high interest. A better understanding of this relationship could potentially lead to the identification of new regulatory pathways and targets for the treatment of bone-related diseases, especially those characterized by BMAT expansion.

Thirdly, effects were documented on bone formation and architecture. In humans, both obesity and T2DM associate with increased fracture risk despite a paradoxically augmented or unaltered BMD, due to low bone turnover and decreased bone quality and strength.^27,31,32^ These conditions also impair fracture healing, with prolonged healing times (delayed union) and a higher incidence of post-fracture complications (such as nonunion or malunion).^10,11^ Strikingly, T2DM patients have even a 30% increased post-fracture mortality compared to non-diabetics.^87^ Several studies in rodents have confirmed this relation between metabolic dysfunction and bone quality deterioration and poor fracture recovery.^21,22,59,88^ However, different from humans, rodent models of obesity with or without overt symptoms of diabetes most commonly show a decrease in trabecular bone mass.^15,59,61,63,89^This was also seen in our results, using the HFD-induced obesity model in male C57BL/6 mice, which is the most widely used rodent model to study obesity.^59^ Yet, while the tibia of the mice on HFD in our study exhibited a modest loss in trabecular bone mass, the impact on trabecular number, thickness, and separation individually was negligible. Others have reported no significant changes or even increased trabecular bone in HFD-fed animals,^14,66^ which likely reflects differences in the susceptibility to HFD of varying mouse strains, ages, and sexes.^60,61,89,90^ In our work, the modest decline in trabecular bone mass in HFD conditions was not recovered by the PHD inhibitor. The administration of FG-4592 rescued the suppressed MAR but not the blocked osteoid deposition. Notably, in the healthy conditions of the ND and in the initial short-term treatment, FG-4592 administration increased the formation of osteoid and its mineralization into bone matrix, resulting in increased trabecular bone mass, particularly in the 2-week treatment cohort. This is in line with ample genetic models of HIF activation in mice, showing its profound capacity to stimulate bone formation and induce high bone mass phenotypes, including by directly activating osteoblast differentiation through Wnt/β-catenin signaling and indirectly by stimulating skeletal vascularization and osteo-angiogenic crosstalk.^45,47,49,50,52,53,91–94^ Surprisingly, the positive effects on bone formation were not associated with a significant impact on the trabecular bone mass after 8 weeks of FG-4592 (Fig. 4j), in contrast to the outcome on the 2-week treatment (Fig. 1h). We presume that the different outcomes with the varying treatment durations might relate to the kinetics of the responses and the relative balances in the bone formation and bone resorption effects locally in the bone tissues, mediated by the osteoblastic and osteoclastic activities, respectively. Our osteoclast-directed analyses, however, did not reveal changes among the groups at the endpoint of the long-term experiment that could clarify why the gain in bone mass had stalled in the 8-week treatment cohort despite the sustained increase in the bone formation parameters. Besides the specifics of the bone formation–resorption dynamics, alternative explanations could be differences depending on the mouse strain or that sustained treatment has diminished efficacy on cancellous bone mass over time. In the cortical bone, minor or no HFD-induced changes were seen within the timespan of our study, as also reported by others.^61,63,89^ The mineral-to-matrix ratio in the diaphyseal cortex tended to be lower in HFD-fed mice, and was rescued by FG-4592, possibly in line with the rescue in MAR. Counterintuitively, cortical porosity was reduced by HFD, yet this was mostly due to a decline in intracortical channels that we speculate to reflect reduced cortical blood vessels. This might reflect a difference between obesity and diabetes, or between mice and humans, as T2DM in humans associates with increased cortical porosity and impaired bone material properties, representing the two key aspects contributing to skeletal frailty and high fracture risk.^27^

Finally, the positive effects on bone of systemic PHD inhibition are obviously also mediated at least in part through the improved control of the overweight and systemic glucose homeostasis, which expectedly ameliorates tissue dysfunction broadly, including in the skeleton. For instance, AGEs, a group of macromolecules formed by non-enzymatic glycation of proteins in conditions of inadequate glycemia control, can accumulate extracellularly in various tissues, affecting their functionality.^27,31,32^ Reduced bone strength, increased fracture risk, and impaired bone repair have all been associated with higher AGEs.^11,28,29^ We found that circulating AGE levels were reduced by PHDi treatment, which could have contributed to the improved bone health. Within the bone itself, however, the AGE levels did not seem to be alleviated by FG-4592. This might relate to their strong binding to collagen and possibly explain why the cortical bone showed little improvement by FG-4592 from the HFD impact.

Given that the HIF-PHD inhibitor was delivered systemically, it could widely target cells and tissues at the organism level, causing HIF activation in bone as well as various other tissues. This is also underscored by the gene expression analyses performed in this work, which provided molecular evidence of successful HIF activation in the body of the FG-4592-treated mice. While a particularly clear readout was obtained in liver samples, the data also supported effective activation of HIF signaling in bone, despite the more restricted and fragmented responses. These might relate to variation and high complexity in the dynamics of the cell- and gene-specific regulatory impact within the heterogeneous bone microenvironment. All in all, numerous and complex tissue-specific HIF-mediated effects in various organs likely contributed to augmenting the metabolic balance by improving overall energy expenditure through enhanced nutrient usage and fat metabolization. For instance, regulatory mechanisms acting in pancreas,^95,96^ gut,^97^ and possibly adipose tissue^98^ could exert additional protective effects in favor of alleviating the symptoms of metabolic dysfunction in systemic PHDi-treated mice. Notably, the bone improvement itself could also contribute to the global metabolism benefits. Bone is a highly active metabolic and endocrine organ that plays critical roles in the regulation of energy metabolism, including through the secretion of osteoblast- and BMAT-derived hormones such as osteocalcin, sclerostin, lipocalin, leptin, and adiponectin.^85,99,100^ In addition, while the skeleton by default already takes up a considerable fraction of circulating glucose,^101^ we previously demonstrated that HIF activation in osteolineage cells further boosts the local consumption and glycolytic turnover of glucose.^49,57^ Indeed, our preceding work revealed that genetic inactivation of VHL in Osx-Cre-targeted osteolineage cells led to excessive glycolytic metabolism and increased overall skeletal glucose consumption, thereby improving global glucose homeostasis and energy metabolism and lowering body weight and peripheral fat accumulation.^49^ Based on this work, we anticipated a joint benefit on bone health and glucose homeostasis by systemic PHDi treatment, as observed here in the context of diet-induced obesity and prediabetic metabolic dysfunction. Taking into consideration all these aspects, it seems most conceivable that the dual beneficial effect of HIF signaling activation on metabolism and bone health and regeneration is driven by a combination of bone-resident HIF responses and systemic contributors.

In conclusion, in this study, we demonstrated the capacity of the HIF-PHD inhibitor FG-4592 to alleviate the skeletal and systemic metabolic consequences of HFD-induced obesity and prediabetes, improving global health as well as recovering many aspects of the overnutrition-associated bone disease and impaired fracture repair. We found the benefits of FG-4592 in bone to be mostly centered on the BM microenvironment, with a marked rescue of HFD-induced defects in medullary vascularization and blocked accumulation of BM adipocytes. Mechanistically, the skeletal benefits of PHD inhibition most likely reflect the net outcome of a combination of direct and indirect impacts of increased HIF activity in bone and beyond, mediating increased bone formation and improved local and global glucose and lipid metabolism. While further work using tissue-specific genetic models will be required to dissect the specific pathways and (either or not bone-resident) HIF-responses involved, through uncovering the joint benefits in an application-directed animal model the current study provides proof-of-concept for the potentially clinically translatable improvement of both metabolism and bone via the use of a single drug. These findings can be the basis for further translational and clinical studies exploring the applicability of this clinically approved, patent-free compound and other HIF-modulating agents in the control of obesity and diabetes and metabolic disorder-related skeletal complications in humans, addressing prime clinical complications relevant to an increasingly large number of people worldwide.

## Materials and methods

Brief outlines of the approaches and methods used are provided here. More detailed descriptions are provided in the Supplementary Information file.

### Animals

WT male CD1 mice were used for the short-term experiments administrating FG-4592 in healthy conditions. C57BL/6J male mice were used for the long-term HFD experiments because of the strong susceptibility of this strain, especially the males, to HFD-induced metabolic dysfunction.^59,60^ High-fat purified diet (HFD) contained 60% of kcal from fat (Research Diets #D12492i). ND control groups were fed with standard grain-based chow (Sniff #V1535-000). The PHD inhibitor FG-4592 (Roxadustat, Med Biochem cat #HY-13426) was delivered i.p. according to the schemes specified for each experiment. DMOG (Cayman) was given i.p. at 100 mg/kg in phosphate-buffered saline, 3 h before euthanasia. For fracture studies, animals were anesthetized with isoflurane, and the left leg was subjected to a full transversal fracture as before,^102^ with the difference that here, no intramedullary stabilization was applied to resemble a compromised healing situation. The animal experiments were reviewed and approved by the KU Leuven Animal Ethical Committee (ECD Projects P041/2017, P042/2017, and P083/2022). More details are provided in the Supplementary Information.

### GTT and indirect calorimetry

For GTT, glucose was injected i.p. at 2 g/kg body weight after overnight fasting. Metabolic rates were measured by indirect calorimetry using automated TSE Phenomaster Calocages, recording food intake, oxygen consumption, carbon dioxide production, and ambulatory activity for a period of 72 h. Heat production and RER were calculated using the recordings from the second 24 h period and corrected for body weight. For details, see Supplementary Information.

### Blood and serum analyses

Blood cell types were measured using an automated SCIL VET ABC PLUS hemocytometer. AGEs were measured using the AGEs assay kit (Abcam #ab273298). CTX levels were determined using the Rat-Laps (CTX-I) EIA assay (Immunodiagnostic Systems). See Supplementary Information for details.

### µCT

Tibias were scanned using a GE nanoCT scanner and analyzed and visualized using software from Bruker, as detailed in the Supplementary Information. Trabecular bone was analyzed in a 1 mm-spanning region of the proximal tibia, excluding the cortex, starting 250 µm (in Fig. 4i) or 1.5 mm (in Fig. 1h) below the growth plate. Cortical bone analyses were performed in a region spanning 0.5 mm of the diaphyseal bone shaft, positioned 2.5 mm below the growth plate. Cortical porosity analyses are described in the Supplementary Information. For fracture calluses, a region of interest (ROI) was set spanning 2.5 mm above and 2.5 mm below the transversal fracture line to segment the callus. The reconstructed 3D µCT scans were used to categorize the union status into bridged or non-bridged, analyzing 8–9 calluses per group (all shown in Fig. S5).

### Thick gelatin histology, IHC, and image analysis

Sample processing, gelatin embedding, immunostaining, and vascular network analysis were done as described in detail by Peredo et al.^103^ and in the Supplementary Information. Femurs were sectioned at 30 µm (for 2D analysis) or 200 µm thickness (for 3D analysis) using a cryostat. Primary antibody (rat anti-EMCN, Santa Cruz #SC65495, 1:200) and secondary antibody (DyLight 550-conjugated donkey anti-rat, diluted 1:500) were incubated for 72 h each, at 4 °C in a humid chamber, and nuclear staining was performed using Hoechst 33342 (20 μg/mL). The samples were cleared and mounted with Rapiclear 1.52 (SUNJinLab) and imaged using a Nikon NiE upright microscope with spinning-disk module (see Supplementary Materials and Methods). 2D vascular analysis was performed on single Z-optical confocal slices, in a 0.8 mm^2^ ROI placed 800 µm below the growth plate (see Fig. 1j), using ImageJ. For 3D image analysis, in a 1.5 × 1.5 mm ROI immediately below the growth plate (see Fig. 3d), images were processed using supervised machine-learning pixel segmentation in Ilastik software and analyzed using ImageJ as detailed in the Supplementary Information.

### Thin section histology, histomorphometry, and FTIRM

Histological processing, staining, and analyses using paraffin and methyl-methacrylate (MMA) sections were performed as described in detail previously^49,102,104^ and in the Supplementary Information. Briefly, fractured tibias dissected at PFD 21 were paraffin-embedded, sectioned at 5 µm, and stained for EMCN (Santa Cruz #SC65495, 1:50) or Safranin O for quantifying callus vascularity or cartilage content, respectively, using ImageJ. Paraffin sections prepared from bones of the mice included in the long-term ND/HFD experiment were stained for osteoclastic tartrate-resistant acid phosphatase (TRAP) activity using 0.5 mg/mL naphthol AS-MX phosphate (Sigma) and 1.1 mg/mL fast red violet LB (Sigma), followed by a fast green counterstain as before.^104^ MMA-embedded tibias were sectioned at 4 µm, stained with toluidine blue, and imaged to quantify adipocytes in a 500 × 500 µm ROI in the proximal metaphysis (see Fig. 3a). Bone histomorphometry was performed according to standardized methods using OsteoMeasure software (OsteoMetrics) as detailed before^104^ and in the Supplementary Information. FTIRM analysis was done on MMA sections of 1 µm thickness as described in the Supplementary Information, measuring 10 fields of 50 × 50 µm each, positioned along the cortex as shown in Fig. 5f.

### Primary osteoblasts

Osteoblasts were isolated from newborn mouse calvaria by digestion with 2 mg/mL collagenase II and 3 mg/mL dispase. Regular culture conditions consisted of 21% O_2_, 5% CO_2_ and 37°C; hypoxic conditions consisted of 1% O_2_, 5% CO_2_ and 37°C. For in vitro application, a stock solution of FG-4592 was prepared in dimethylsulfoxide (DMSO) at 50 mmol/L and diluted in αMEM for use. Non-stimulated cells received equal volumes of DMSO (vehicle). For details, see Supplementary Information.

### qRT-PCR and Western blot

RNA extraction from bones and cultured cells, and subsequent cDNA synthesis, are described in the Supplementary Information. Gene expression levels were measured on a Step-One-Plus Real-Time PCR system using Fast SYBR™ Green Master Mix (Applied Biosystems/ThermoFisher Scientific) and quantified by the delta-Ct method (2^−ddCt^) relative to the expression of a housekeeping gene. Primer sequences are supplied in Supplementary Table S1. Protein extraction and Western blot were performed by standard methods, detailed in the Supplementary Information, including the antibodies and detection reagents used. Unedited gel and blot images are provided in Fig. S6.

### Statistics

All graphical data are shown as mean ± SD, with individual independent data points shown on the bar plots except for longitudinal data (GTT, body weight follow-up) and frequency distribution plots. The sample sizes were determined based on previous experiments characterizing bone and metabolic phenotypes in young adult mice performed in our lab,^49,102,104^ and are indicated in the figure legends for each experiment. Outliers were excluded using the ROUT (*Q* = 2%) method. Statistical analyses were done by *t*-tests when comparing two groups (vehicle versus treatment), one-way ANOVA when comparing one variable among more than two groups, and two-way ANOVA when comparing 2 variables, which in this work were diet (ND versus HFD) and treatment (vehicle versus FG-4592/Roxadustat), with interaction between the variables indicated in the legends. Multiple comparisons were done with Tukey’s post hoc test. *P* values < 0.05 (two-tailed) were considered statistically significant. For statistical analyses and preparing graphs, we used GraphPad Prism (v10.2.3).

## Supplementary information

Supplementary Information

## Data Availability

The numerical data are transparently shown in the graphs. The imaging data underlying the fracture bridging analysis and unedited full Western blot and gel images are supplied in the Supplementary Information. All other data related to the presented results will be provided by the responsible author upon reasonable request.
