# Supplementary material for: Pharmacological HIF activation protects against diet-induced obesity, glucose intolerance, and skeletal dysfunction by exerting dual beneficial effects on energy metabolism and bone
Source: Bone Res. 2026 Feb 11;14:21. doi: 10.1038/s41413-025-00503-3 (PMC12891525; doi:10.1038/s41413-025-00503-3)
Supplement: Supplementary file 1 — Supplementary Information [file 41413_2025_503_MOESM1_ESM.pdf]

Supplementary Information

This file contains:

Supplementary Materials and Methods

Supplementary Table S1

Supplementary Figures 1-6

Supplementary References

Pharmacological HIF activation protects against diet-induced obesity, glucose intolerance and skeletal dysfunction by exerting dual beneficial effects on energy metabolism and bone

Roger Valle-Tenney[1], Nicolas Peredo[1,4], Karen De Samblancx[1], Elena Nefyodova[1], Ruben Cardoen[1], Tom Dehaemers[1], Delphine Farlay[2], Roland Chapurlat[2], Bart Van der Schueren[3], Chantal Mathieu[3], Roman Vangoitsenhoven[3], and Christa Maes[1,*]

[1] Laboratory of Skeletal Cell Biology and Physiology (SCEBP), Skeletal Biology and Engineering Research Center (SBE), Department of Development and Regeneration, KU Leuven, Leuven, Belgium.
[2] INSERM, UMR 1033, Univ Lyon, Université Claude Bernard Lyon 1, F-69008 Lyon, France.
[3] Department of Endocrinology, University Hospitals Leuven, and Department of Chronic Diseases and Metabolism, KU Leuven, Leuven, Belgium.
[4] Present address: VIB BioImaging Core Leuven, Center for Brain and Disease Research, KU Leuven, Leuven, Belgium.

* Corresponding author: Prof. Dr. Christa Maes, christa.maes@kuleuven.be

Supplementary Materials and Methods

Animal models and treatments

WT male CD1 mice were initially used to validate the activity of the FG4592 compound *in vivo* and for exploring the potential of the drug to elicit effects systemically and in bone, in short-term experiments administrating FG-4592 in healthy conditions. As the initial data led us to speculate that the treatment could have benefits in settings reflecting obesity and prediabetes, the long-term experiments in high-fat diet (HFD) conditions were initiated. For these experiments, C57BL/6J mice were used, because of the strong susceptibility of this strain to HFD-induced metabolic dysfunction (BW change and glucose intolerance) compared with other strains.[1] This has led the C57BL/6J strain to represent one of the best-studied models of diet-induced obesity, with high sensitivity to the metabolic stress as well as documented adverse effects on trabecular bone.[2] Male C57BL/6J mice were used, because HFD was reported to induce obesity and metabolic dysfunction more strongly in males, and to associate with a greater cancellous bone loss in male than female mice.[3,4] The mice were obtained from the KU Leuven Animal Facility, where the C57BL/6J strain is bred after having been sourced originally from The Jackson Laboratory via Charles River Laboratories.

Animals on HFD regimen received a high-fat purified diet containing 60% of kcal from fat (Research Diets #D12492i), refreshed weekly. Normal diet (ND) control groups were fed with standard grain-based chow (Sniff #V1535-000). Body weight of animals was followed up once a week. The PHD inhibitor FG-4592 (Roxadustat, Med Biochem cat# HY-13426) was resuspended to 50 mg/mL in 5% glucose solution supplemented with 3 µL of 1N NaOH per mg of Roxadustat, and delivered i.p. according to the schemes as specified for each experiment. DMOG (Cayman) was given i.p. at 100 mg/kg in phosphate-buffered saline (PBS), 3 h prior to euthanasia. For fracture model studies, analgesia (Buprenorphine 0.2 mg/kg s.c.) was administered 2 h prior to the surgery, and twice daily up to 3 days post-fracture. Animals were anesthetized with isoflurane (2% in 1L of O_2/min) during the procedure and ocular drops (DuraTears) were applied to prevent dry eyes. The left leg was shaved and a 0.5 to 1 mm

incision was made to expose the tibia, as described before.[5] A transversal full fracture was induced, approximately 1 cm distal from the knee, using an electric mini saw (Dremel) loaded with a high-flex diamond mini disc (6.5 mm Ø, 0.15 mm thickness) (Komet Dental #943104065) at maximum speed. No stabilization (e.g., by means of an external fixator or an intramedullary pin) was applied in this model. The rationale for using a non-stabilized fracture model was that the healing process is more challenged and extended in time, especially in HFD-fed mice (see Figure 6h), thereby reflecting a compromised fracture repair situation and offering a fitting model for testing pro-regenerative interventions under compromised healing conditions. The skin was closed using 6-0 braided absorbable silk suture and cleaned with chlorhexidine 0.2% to prevent infections. Animals were recovered on a heating pad for around 15 min to prevent hypothermia and housed in a pre-heated clean cage with easy access to food pellets.

Glucose tolerance tests (GTT) and indirect calorimetry

For GTT, animals were fasted overnight and blood glucose levels were measured using a OneTouch Verio glucose monitor (LifeScan). Glucose was injected i.p. at 2 g/kg body weight and monitored every 15 and 30 min up to 3 h. Metabolic rates were measured by indirect calorimetry using automated TSE Phenomaster Calocages. Mice were acclimatized to the specific drinking and feeding instruments and the individual housing for 3 days prior to the measurements. In the calocages, an ambient temperature of 22°C was maintained under a 12-h dark/light cycle. Mice had ad libitum access to food and water. Food intake, oxygen consumption, carbon dioxide production, and ambulatory activity were monitored for a total period of 72 h. Heat production and respiratory exchange ratio (RER) were calculated for each mouse using the recordings from the second 24 h period and corrected for body weight.

Blood and serum analyses

Around 500 µL of blood was collected by cardiac puncture after euthanasia. A fraction of the sample was used for measuring the blood cell types using an automated SCIL VET ABC PLUS hemocytometer. Serum was obtained after coagulation at room temperature for 1 h, followed by centrifugation at 1,000 g for 10 min. AGEs were measured using the Advanced Glycation End Products assay kit (Abcam #ab273298), applying 700 µg protein per sample, according to the manufacturer's instructions. CTX levels were determined using the Rat-Laps (CTX-I) EIA assay (Immunodiagnostic Systems) according to the instructions of the kit, using serum of mice that was harvested after 3 h starvation to avoid interference of food intake on the measurement of the CTX-I collagen degradation products.

Micro-computed tomography (µCT)

Tibias were scanned using the GE nanoCT scanner (at 50 kV, 200 mA, 5 µm voxel size) and reconstructions and analyses of the scans were performed using Nrecon and Ctan software (Bruker). 3D images of tibias and fracture calluses were generated using CTVox (Bruker). Trabecular bone analyses were performed in a 1 mm-spanning region of the proximal tibia excluding the cortex, starting 250 µm (in Fig. 4j) or 1.5 mm (in Fig. 1h) below the growth plate to exclude the primary spongiosa. Trabecular bone volume over tissue volume (BV/TV), trabecular number (Tb.N), trabecular thickness (Tb.Th) and trabecular separation (Tb.Sp) were determined. Cortical bone analyses were performed in a region spanning 0.5 mm of the diaphyseal bone shaft, positioned 2.5 mm below the growth plate. Cortical thickness (Ct.Th), periosteal bone perimeter (B.Pm), endocortical perimeter (Ec.Pm) and medullary area (M.Ar) were calculated in CTan. For cortical porosity analyses, the cortex of the tibia was selected from the bottom of the growth plate to 8 mm distal, and subsequently segmented into mineralized and non-mineralized pixels. The non-mineralized intracortical space was subsequently segmented by size into lacunar porosity (<8µm Ø) and intracortical channels

(>8μm Ø). Total porosity, lacunar porosity and intracortical channels volume % were calculated relative to the cortical volume. For fracture calluses, a region of interest (ROI) was set spanning 2.5 mm above and 2.5 mm below the transversal fracture line to segment the callus. The reconstructed 3D μCT scans were used to categorize the union status into bridged or non-bridged, analyzing n=8-9 samples per group.

Thick gelatin sectioning, immunohistochemistry (IHC), image acquisition and vascular network analysis

Sample processing, embedding, immunostaining and network analysis were described in detail in Peredo et al (2022)[6]. Briefly, after dissection, bones were fixed in freshly prepared 4% paraformaldehyde in PBS overnight. Samples were washed 3 times with PBS and decalcified in 0.5 M ethylenediamine tetraacetic acid (EDTA) solution, replaced 3 times per week, for 2 weeks. Gelatin-embedded femora were sectioned at 30 μm (for 2D analysis) or at 200 μm thickness (for 3D analysis) using a cryostat and collected on SuperfrostPlus slides (Epredia #J1800AMNZ). Gelatin sections were stored at -80°C until use. Prior to the immunostaining protocol, the sections were thawed and dried at room temperature for 45 min to ensure adherence, and enclosed by a hydrophobic draw. Samples were rehydrated with PBS for 15 min, permeabilized with PBS-Tween 0.1% + Triton-100 0.3% for 1 h and blocked overnight in permeabilizing solution supplemented with serum from the host species in which the secondary antibody was raised. Primary antibody (rat anti-EMCN, Santa Cruz #SC65495, 1:200) was diluted in permeabilizing solution and incubated 72 h at 4°C in a humid chamber. Three consecutive washes with PBS-Tween 0.1% were performed (5 min each). Secondary antibody (DyLight 550-conjugated donkey anti-rat, diluted 1:500) was incubated in PBS-Tween 0.1% during 72 h in a humid chamber. Nuclear staining was performed using Hoechst 33342 diluted 1:500 in PBS (20 μg/ml) during 1 h at room temperature, followed by 3 PBS-Tween 0.1% washing steps. Finally, the samples were cleared and mounted with Rapiclear 1.52 (SUNJinLab).

Images were acquired using a Nikon NiE upright microscope equipped with a Yokogawa CSU-X spinning-disk module with Prime BSI camera (Teledyne Photometrics) combined with a multi-immersion 20X Plan Fluor MImm DIC N2 objective (NA 0.75) (Nikon). Large image tiles were acquired using a 0.6x digital zoom, imaging up to a depth of 100 µm using 1-µm Z-steps, and stitched using NIS Elements (Nikon). Hoechst was excited with a 405 laser (violet cube 100mW) and collect with a 595/50 emission filter, DyLight 550 was excited with a 561 laser (Green diode-sapphire 50mW) and collected with 450/50 emission filter.

2D vascular analysis was performed on single Z-optical confocal slices, in a 0.8 mm^2 ROI placed 800 µm below the growth plate (see Fig. 1j). EMCN-stained blood vessels were manually segmented, and morphological parameters (vascularized area and local vessel thickness) were calculated using ImageJ. For 3D image analysis, a 1.5 x 1.5 mm ROI located immediately below the growth plate was selected (see Fig. 3d). Images were pre-processed using supervised machine-learning pixel segmentation in Ilastik software[7] and cleaned from unspecific noisy particles by excluding elements smaller than 0.45 micron in diameter using the 'Particle size 3D volume' ImageJ plugin from MorpholibJ[8]. Binarized images depicting blood vessel walls stained for EMCN were visually explored in parallel to the original images to manually fill the lumens and obtain the skeletonized renderings of the vascular networks, which were used to calculate the networks' topological parameters in ImageJ as described in Peredo et al. (2022).[6] The quantifications were normalized to the total BM tissue volume, calculated based on the Hoechst nuclear signal channel by applying 3D distance dilation, erosion and filling functions from ImageJ, and corrected to exclude empty regions.

Paraffin histology of fractured bones

Our lab's histological processing, staining and analyses methods using paraffin sections from mouse bones have been described in detail previously.[5,9,10] Briefly, fractured tibias were dissected at PFD 21, fixed overnight using freshly prepared 2% paraformaldehyde at 4°C, decalcified in 0.5 M EDTA for 2 weeks, embedded in paraffin, and sectioned at 5 µm. To visualize the bone vasculature, sections were subjected to IHC for EMCN (rat anti-EMCN, Santa Cruz #SC65495, 1:50), detected using biotin-conjugated goat-anti rat secondary antibodies and the TSA Indirect Biotin amplification System (PerkinElmer), with streptavidin-HRP and DAB-based visualization for brightfield detection. Images were acquired using an Olympus IX83 inverted microscope equipped with a DP73 camera. Blood vessels were manually drawn in the corresponding ROI, and area and thickness were quantified using ImageJ software. To visualize cartilage proteoglycans, sections were stained with Safranin O and counterstained with Fast Green to visualize the tissue. Images were acquired on the Olympus IX83, and the cartilage fraction present in the calluses was analyzed by manually drawing the callus tissue as ROI, followed by segmentation of the red Safranin O signal in ImageJ using Color Thresholding. The cartilage fraction relative to the callus tissue area was quantified on 4 sections per bone, to generate a representative average value for each animal, and a total of 8 animals were analyzed per group.

Paraffin and methyl-methacrylate (MMA) histology and standard bone histomorphometry for analysis of bone remodeling

Full tibias from mice included in the long-term ND/HFD experiment were dissected, fixed and decalcified as described above, and 5 µm paraffin sections were stained for TRAP based on its enzymatic activity. The contralateral tibias were fixed in Burckhardt's solution (~12% formalin in 64% methanol and glucose-phosphate buffer), rinsed with 100% ethanol, embedded (non-decalcified) in MMA and sectioned at 4µm. MMA sections were stained with

toluidine blue and imaged using an Olympus IX83 microscope with DP73 camera to visualize BM adipocytes. Adiposity analyses were performed by manually drawing adipocytes in a 500 x 500 µm ROI placed in the proximal tibial metaphysis (see Fig. 3a). Area and number were calculated in ImageJ. Bone histomorphometry was done according to standardized methods as before.[9] For dynamic histomorphometry, calcein (20 mg/kg BW) was injected 1 and 4 days before euthanasia and the labels were quantified in the trabecular bone in a 1 mm^2 proximal tibia ROI. Mineral apposition rate (MAR, µm/day) and mineralized surface were corrected for bone surface, and bone formation rate (BFR) was corrected for bone area ($µm^3/µm^2$/day) using OsteoMeasure software (OsteoMetrics). For static histomorphometry, TRAP-stained sections were used to count TRAP+ osteoclasts on the trabecular bone surface (expressed as osteoclast surface over bone surface (OC.S/BS, in %) and osteoclast number relative to bone perimeter (N.Oc/B.Pm, #/mm)), and MMA sections stained with Von Kossa/Van Gieson were used to quantify the osteoid matrix (as osteoid surface (OS) normalized to trabecular bone surface (BS)), all in a 1 mm^2 square ROI positioned in the proximal metaphysis at 250 µm below the edge of the growth plate, using OsteoMeasure software. Three to four sections were measured per bone to obtain representative average values for each animal, and n=7-9 animals were analyzed per group.

Fourier-transform infrared microspectroscopy (FTIRM)

FTIRM analysis was performed with a Spotlight 200i MCT Spectrum 3 DTC System (Perkin-Elmer) equipped with wide-band detector. The spectra were recorded from 4,000 to 400 cm^{-1} at a spectral resolution of 4 cm^{-1}. For each sample, 3 MMA-embedded tibia sections of 1 µm thickness were analyzed in transmission mode. Measurements were performed in 10 ROI fields of 50 x 50 µm each, positioned along the cortex from the proximal region (cortex adjacent to the metaphysis) to the diaphysis (see Fig. 5f), and averaged for each compartment. Contribution of air and MMA were subtracted from the raw spectra, and curve-fitted with Python software as before.[11] Five variables were analyzed: mineral/matrix ratio (v_1v_3

PO_4/amide I area ratio), mineral maturity (1030/1110 cm^{-1} area ratio) reflecting the transformation of mineral precursors into apatite crystals, mineral crystallinity index (1/full width at half maximum of the peak at 604 cm^{-1} from v_4 PO_4 = apatitic PO_4) assessing crystal size/perfection, mineral carbonation (measuring the incorporation of carbonates onto bone mineral, v_2 CO_3/v_1v_3 PO_4 area ratio), and collagen maturity (1660/1690 cm^{-1} area ratio) measured using amide I domain (also see Supplementary Fig. S3), all as before.[11-13]

Primary osteoblasts (pOBs)

pOBs were isolated from newborn mouse calvaria. After dissection, the calvaria were briefly rinsed in 70% ethanol and sterile PBS, pooled per 3, and kept in FBS-free αMEM with penicillin-streptomycin (GIBCO) until digestion with 2 mg/ml collagenase II and 3 mg/ml dispase in FBS-free αMEM medium (all from Gibco). The supernatant of the first 10-min digestion was discarded, and those of the five subsequent digestion steps (15 min each, with gentle shaking, at 37°C) were collected in αMEM containing 10% FBS. Afterwards, cells were centrifuged 8 mins at 500g and resuspended in αMEM with 10% FBS, 1% penicillin-streptomycin, and 2.5mM ascorbic acid. Cells were seeded in T75 flasks, washed with PBS the next day to remove non-adherent cells, and cultured in αMEM with 10% FBS and penicillin-streptomycin for 3-5 days until reaching 70% confluency. Regular culture conditions consisted of 21% O_2, 5% CO_2 and 37°C; hypoxic conditions consisted of 1% O_2, 5% CO_2 and 37°C. For in vitro application, a stock solution of FG-4592 was prepared in dimethylsulfoxide (DMSO) at 50 mM and diluted in αMEM for treatment. Non-stimulated cells received equal volumes of DMSO (vehicle).

RNA extraction and qRT-PCR

Total RNA was isolated from bones by crushing the tissues in a porcelain mortar with liquid nitrogen and Trizol (Invitrogen), according to the manufacturer's instructions. For RNA extraction from cultured cells, lysis buffer (1% 2-ME in RLT buffer, Qiagen) was added and the cells were scraped off the plates. RNA was extracted with the RNeasy minikit (Qiagen) following the manufacturer's instructions. The RNA concentration was determined by Nanodrop spectrometry (ThermoFisher Scientific) and integrity was checked using the bleach gel method. cDNA was synthesized from 500ng RNA using Superscript RT (SII, Invitrogen). Gene expression levels were measured on a Step-One-Plus Real-Time PCR system using Fast SYBR™ Green Master Mix (Applied Biosystems/ThermoFisher Scientific) and quantified using the delta-Ct method (2^-ddCt) relative to the housekeeping gene *hypoxanthine phosphoribosyltransferase* (*Hprt*) or in selected cases *18S* ribosomal RNA as indicated in the legends. The primer sequences used are provided below, in Supplementary Table S1.

Protein extraction and Western blot

Proteins from tibias, humeri, and liver samples were extracted in cell extraction buffer (CEB) supplemented with proteinase and phosphatase inhibitors PMSF (0.03 mM), sodium fluoride (NaF, 5mM), sodium orthovanadate (Na3VO4, 0.25 mM) and protease inhibitor cocktail (cOmplete, Roche). Tissues were homogenized in a porcelain mortar with liquid nitrogen and sonicated for 10 seconds on ice. Samples were centrifuged for 10 min at 14,000 rpm at 4°C to pellet the insoluble material. Protein concentrations were determined using the Bradford assay (Bio-Rad Laboratories). The samples were loaded onto a 4-10% Bis-Tris protein gel (NuPAGE Novex). Proteins were transferred from SDS-PAGE gels into PVDF membranes (Immobilon-P Millipore #IPVH00010) in a semi-dry transfer machine (Trans-Blot, Biorad). Standard western blotting procedures were used, with primary antibodies against HIF-1α (Calbiochem #10006421), GAPDH (ThermoFisher #AM4300), β-actin (SantaCruz #sc-

47778), AGEs (Abcam #ab23722) and Histone H3 (Abcam #ab47287), and HRP-conjugated secondary antibodies. For detection, the blots were incubated with chemiluminescent substrate (Supersignal West Dura or West Femto substrates, ThermoFisher Scientific) and visualized with a chemiluminescence imager (GE Amersham Imager 600). Unedited gel and blot images are provided in Supplemental Fig. S6.

Supplementary Table S1. Primer sequences used for qRT-PCR analysis.

Gene	Forward primer sequence	Reverse primer sequence
18S	GCA ATT ATT CCC CAT GAA CG	GGC CTC ACT AAA CCA TCC AA
Egln1(Phd2)	TTG TTA CCC AGG CAA CGG AAC	CCT TGG CGT CCC AGT CTT T
Epo	CAT CTG CGA CAG TCG AGT TCT G	CAC AAC CCA TCG TGA CAT TTT C
Glut1	TAT TGC CCA GGT GTT TGG CT	TCC CTC GAA GCT TCT TCA GC
Hprt	TGC TGA CCT GCT GGA TTA CA	TAT GTC CCC CGT TGA CTG AT
Ldha	TGT CTC CAG CAA AGA CTA CTG T	GAC TGT ACT TGA CAA TGT TGG GA
Pdk1	CCC CGA TTC AGG TTC ACG	CCC GGT CAC TCA TCT TCA CA
Pgk1	TGG TGG GTG TGA ATC TGC C	ACT TTA GCG CCT CCC AAG ATA
Vegf	AGT CCC ATG AAG TGA TCA AGT TCA	ATC CGC ATG ATC TGC ATG G

Supplementary Figures

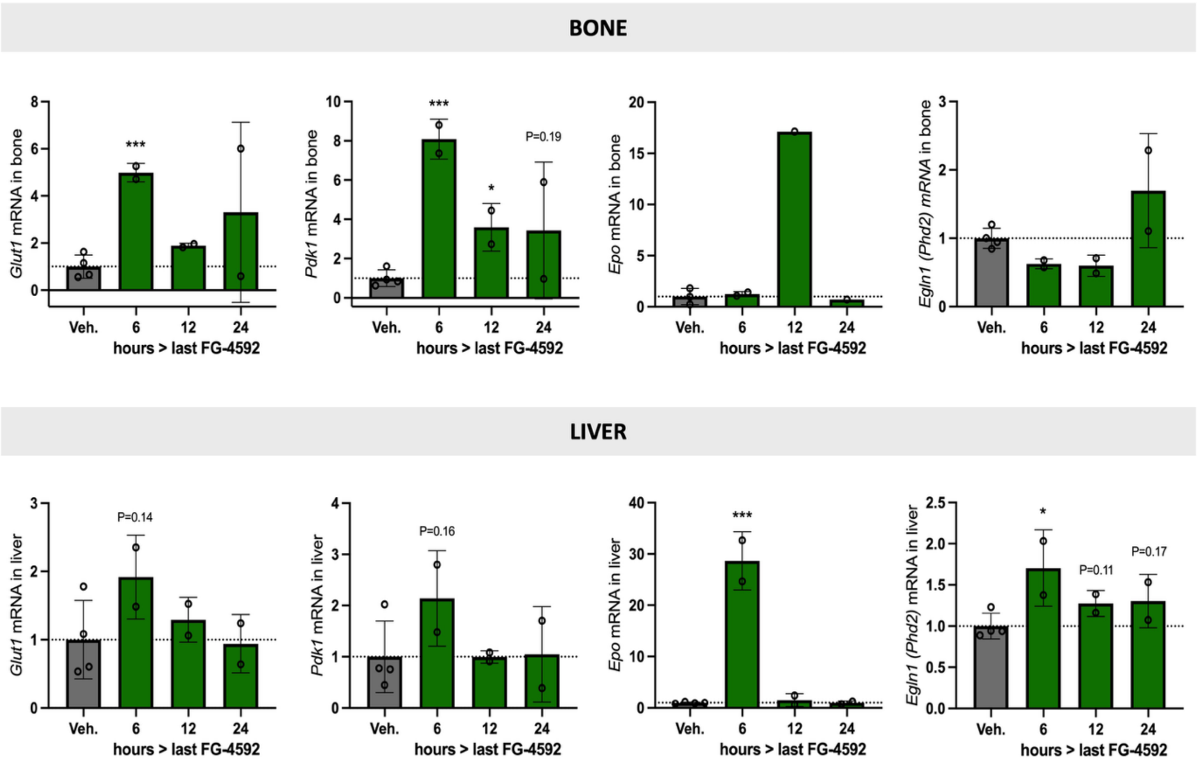

Supplementary Fig. S1. HIF target gene expression after FG-4592 administration *in vivo.* qRT-PCR assessment of the expression levels of HIF-regulated genes *Glut1, Pdk1, Epo,* and *Egln1 (Phd2)* in (top panels) bone samples and (bottom panels) liver tissue samples harvested from healthy (regular chow-fed) 12-week-old CD1 mice that had received a total of seven i.p. injections with FG-4592 (Roxadustat) over a period of 2 weeks (according to the scheme indicated in Fig. 1f), with the last injection received 6, 12, or 24 hours prior to euthanasia. Bone samples represented femoral cortical shafts flushed to eliminate the bone marrow. Gene expression was calculated relative to the housekeeping genes *18S* (for *Glut1* and *Pdk1*) or *Hprt* (for *Epo* and *Egln1*) and normalized to the expression levels in the corresponding tissue samples from vehicle-treated animals. Notwithstanding the experimental nature and small sample size in this explorative validation setup, statistical analysis was applied on the data, showing the results of t-testing between vehicle-treated mice (n=4) and FG-4592-treated mice (n=2 per time point). *P<0.05, ***P<0.001.

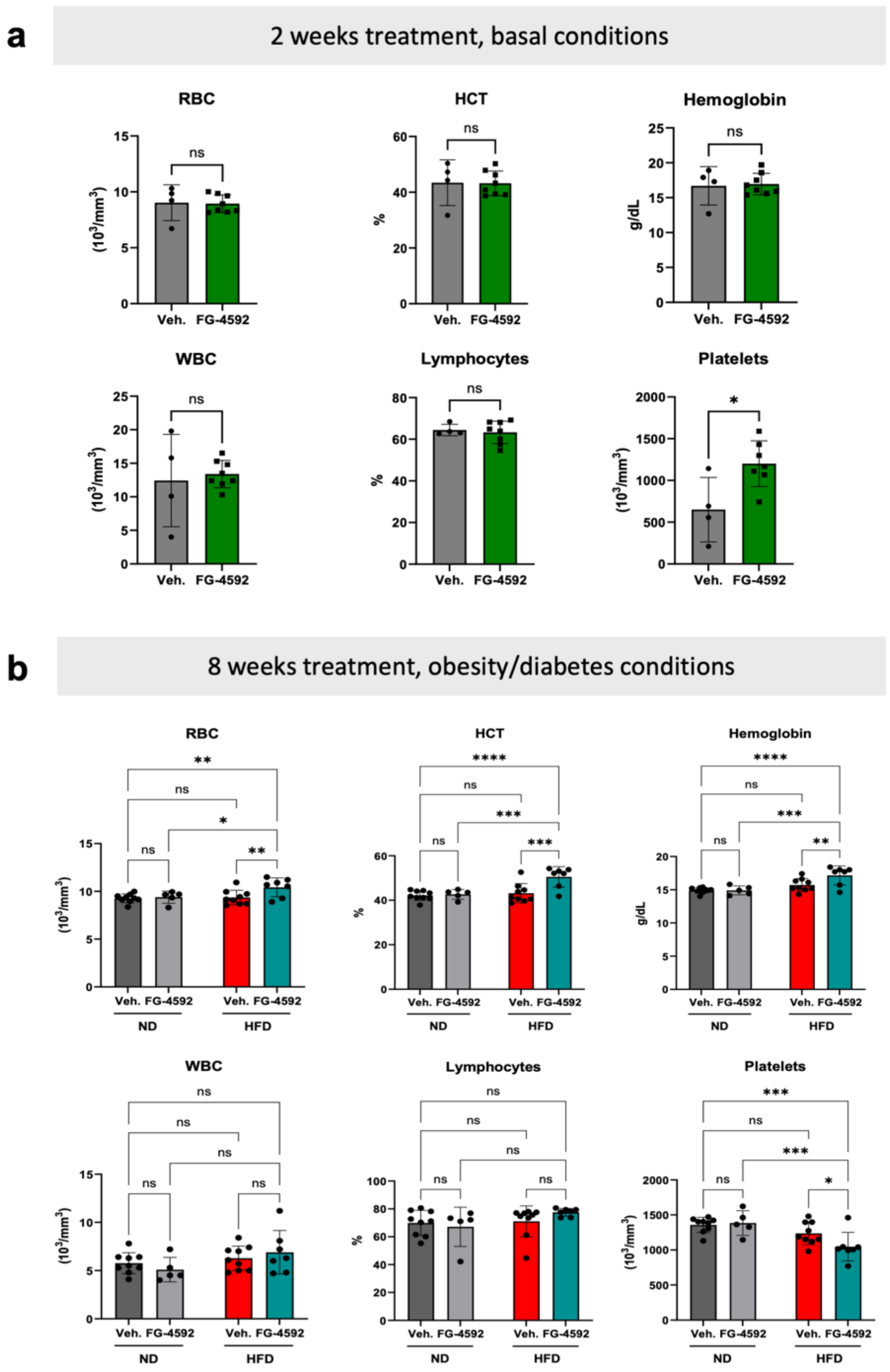

Supplementary Fig. S2. Hematologic analysis of blood cell types.

a-b Circulating blood cell types as quantified for the experimental mice included in this study, showing red blood cells (RBC), hematocrit (HCT), hemoglobin, white blood cells (WBC), lymphocytes and platelets, for (**a**) the experiment outlined in Fig. 1f, assayed after two weeks of vehicle versus FG-4592 treatment in basal/healthy conditions; and (**b**) the experiment outlined in Fig. 2a, including mice on normal diet (ND) versus high-fat diet (HFD) and treated with vehicle or FG-4592 for 8 weeks. Statistical analysis in (**a**) was done by t-test, and in (**b**) by two-way ANOVA with multiple comparisons; significances are shown as *P<0.05; **P<0.01; ***P<0.001; ****P<0.0001; ns, non-significant.

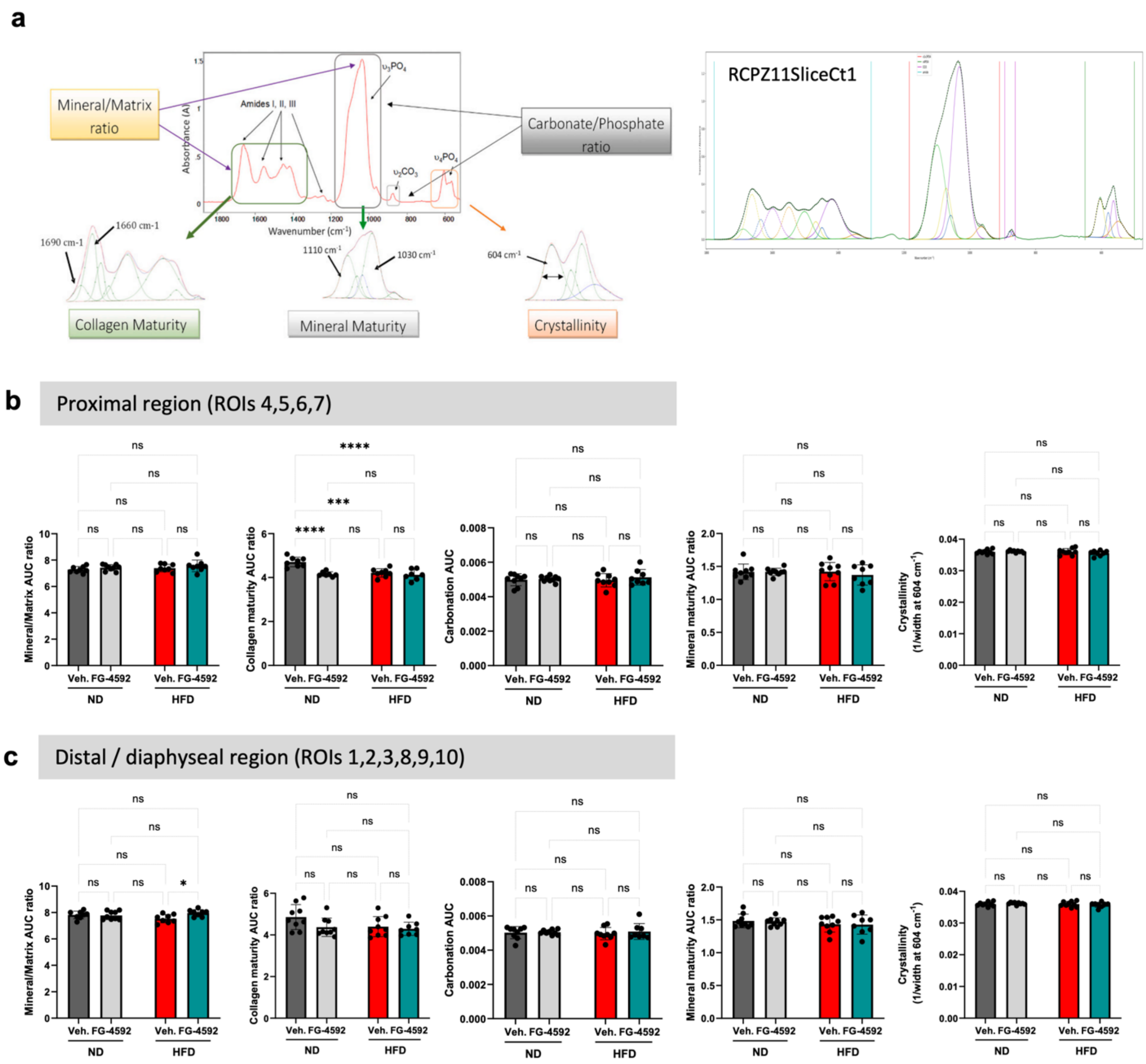

Supplementary Fig. S3. Fourier-transform infrared micro-spectroscopy (FTIRM).

a Infrared spectral overview of peaks depicting Amides (I, II, III), PO_4, CO_3, and spectral fitting for mineral/matrix ratio, carbonate/phosphate ratio, collagen maturity, mineral maturity, and crystallinity calculations (obtained from[14]). Representative image of the spectral pre-treatment (multilinear baseline correction, MMA removal, smoothing) using the previously reported custom fitting algorithm.[11]

b-c Mineral/matrix ratio, collagen maturity, carbonation, mineral maturity, and crystallinity of the cortical bone (**b**) adjacent to the metaphyseal region of the proximal tibia, and (**c**) in the more distal diaphyseal region, as measured in samples from mice included in the experiment outlined in Fig. 2a, exposed to ND versus HFD for 16 weeks, and treated with FG-4592 versus vehicle during the last 8 weeks. Statistical analyses were done by two-way ANOVA, with multiple comparisons between all the groups, shown as *P<0.05; ***P<0.001; ****P<0.0001; ns, not significant.

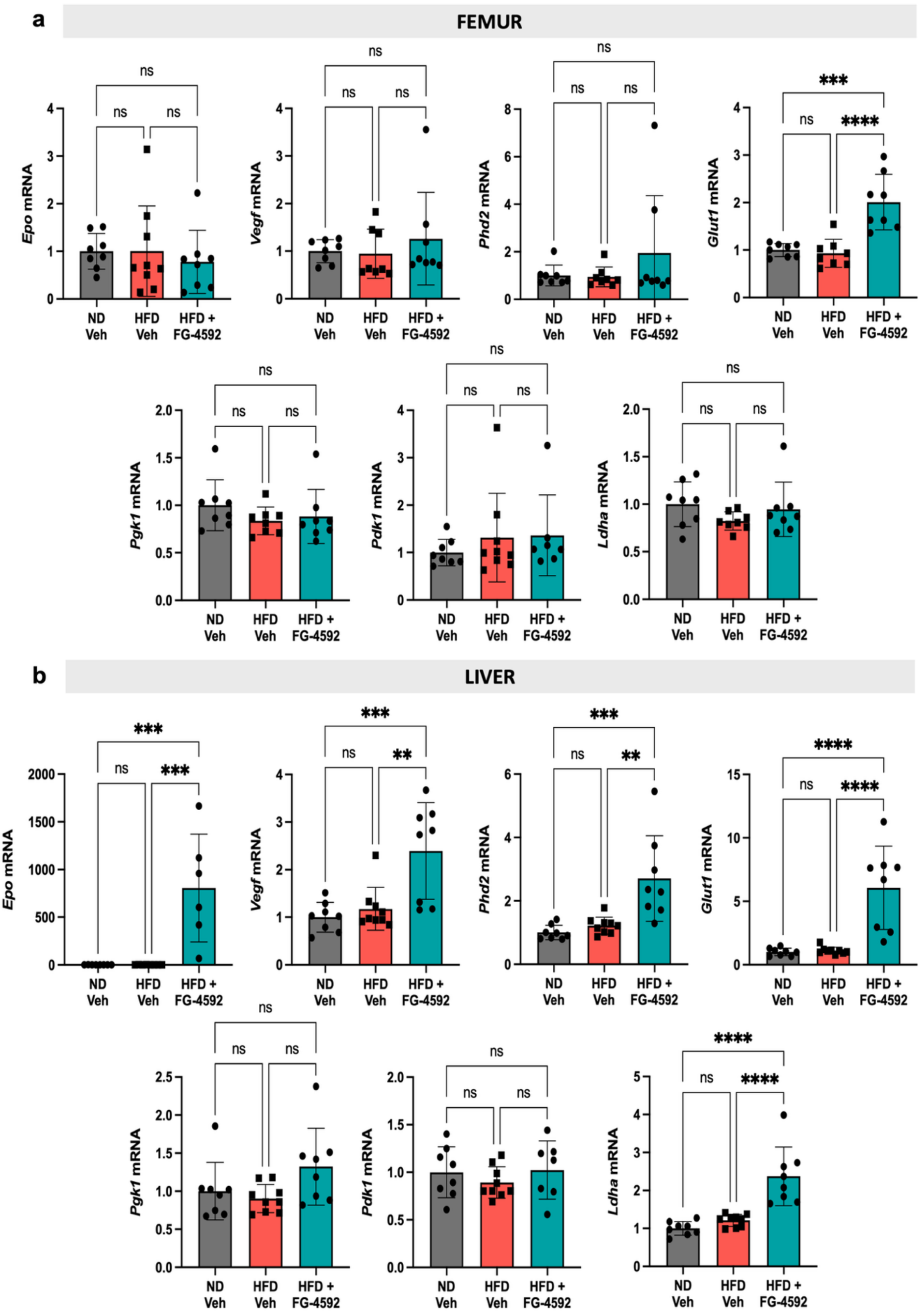

Supplementary Fig. S4. Legend, see next page.

Supplementary Fig. S4. HIF target gene expression after FG-4592 administration in metabolically challenged mice.

a-b qRT-PCR assessment of the expression levels of HIF-regulated genes *Epo, Vegf, Egln1 (Phd2), Glut1, Pgk1,Pdk1,* and *Ldha* in (**a**) full femurs and (**b**) liver tissue samples harvested from 27-week-old male C57BL/6J mice that had been fed HFD from 8 weeks of age onward, and received a total of 10 i.p. injections with FG-4592 (Roxadustat) over a period of 3 weeks during which they recovered from a tibia fracture (according to the scheme indicated in Fig. 6a), with the last injection received 1 h prior to euthanasia. Gene expression analyses were performed on the femurs from the contralateral (uninjured) leg and on liver tissues, calculated by the delta-delta-Ct method relative to the housekeeping gene *Hprt*, and normalized to the expression levels in the corresponding tissue samples from vehicle-treated animals fed regular chow (normal diet, ND). Statistical analysis was performed by 1-way-ANOVA across the three experimental groups, with significant results indicated as *P<0.05, **P<0.01, and ***P<0.001, and non-significant comparisons marked as 'ns'.

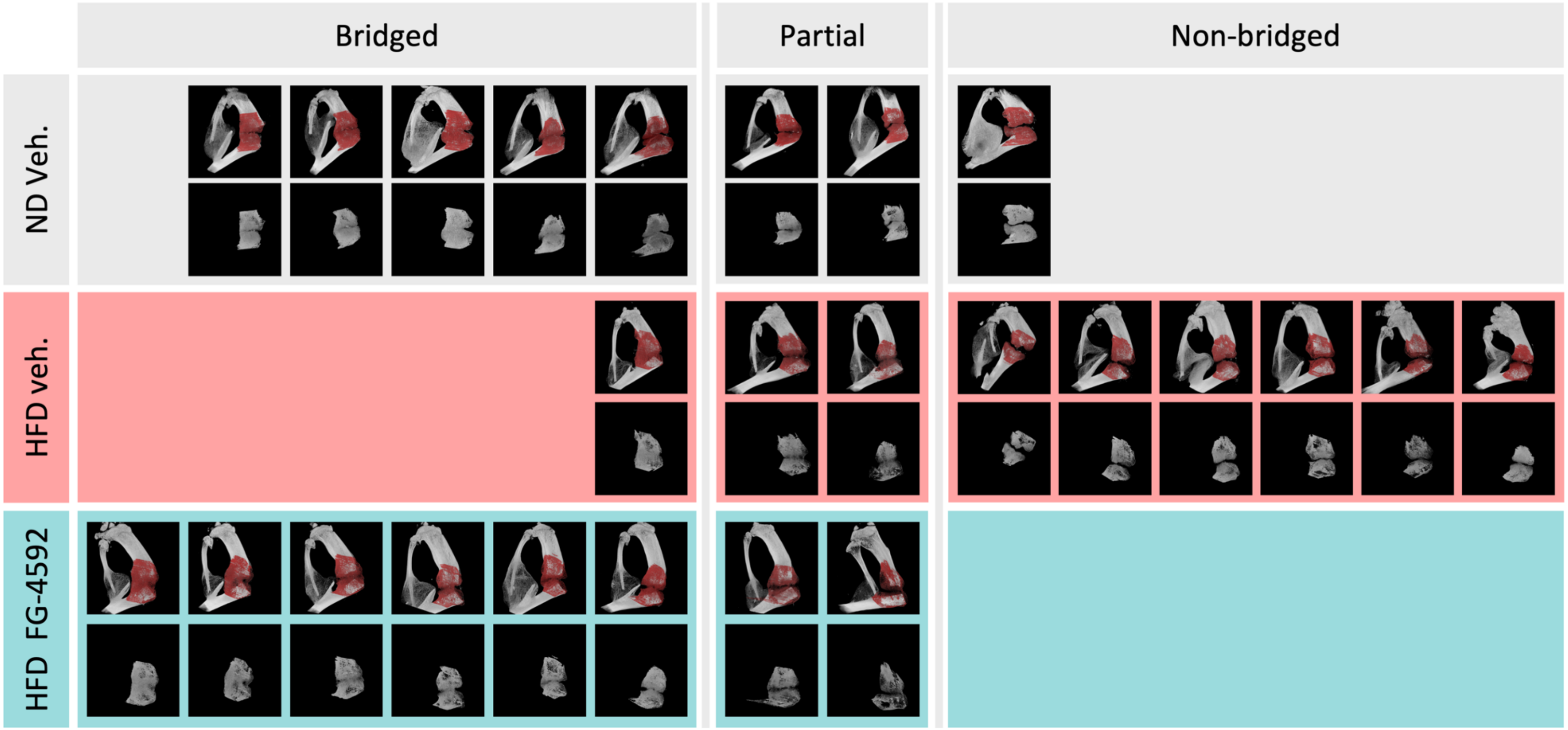

Supplementary Fig. S5. Overview of non-stabilized tibia fractures.

3D reconstructions of µCT scans generated at post-fracture day (PFD) 21 from non-stabilized tibia fractures of mice subjected to ND or HFD regimen for 16 weeks, and treated with FG-4592 (20 mg/kg i.p.) or vehicle 3 times per week from PFD 2 until PFD 21. Newly formed callus tissue was segmented in red pseudo-colour. The callus union status was categorized as (i) bridged, (ii) partially bridged and (iii) non-bridged based on the mineralized tissue present at the fracture line.

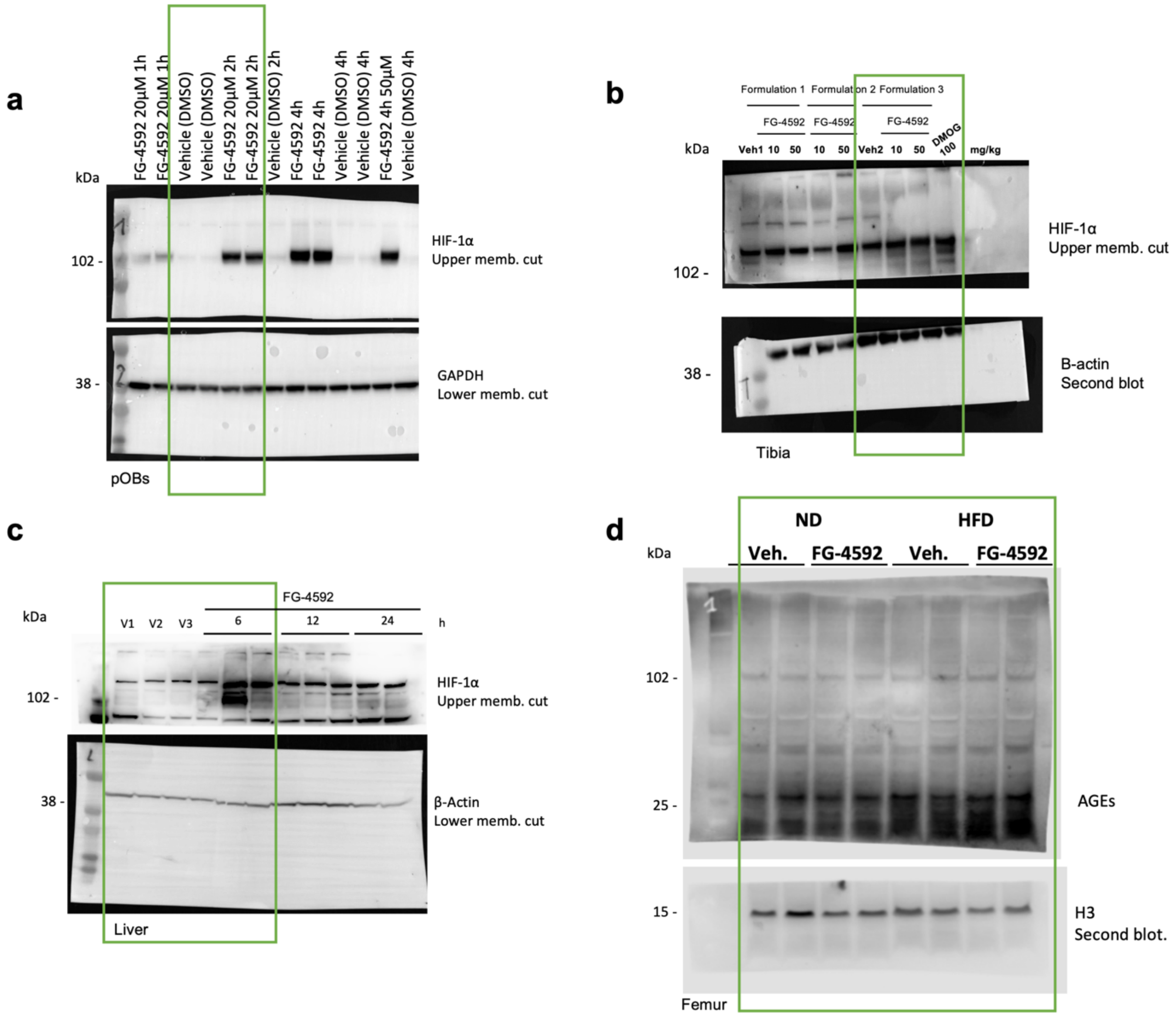

Supplementary Fig. S6. Raw Western blot images.

a-d Full unedited immunoblots, highlighted the cropped lanes that are shown in the main manuscript's figure panels by green rectangles. (**a**) Full unedited gel for Fig. 1a, showing samples of pOBs treated with FG-4592 (20 µM) or vehicle (DMSO) for 2 or 4 h. Each lane represents a technical replicate, with 1 or 2 replicates loaded per condition. The membrane was cut into 2 pieces to incubate anti-HIF1α and anti-GAPDH antibodies, respectively. (**b**) Full unedited gels for Fig. 1d. Tibia protein extracts of mice treated with FG-4592 at different concentrations and diluted in 3 different formulations, harvested 3 h after i.p. injection. Formulation 3 uses glucose+NaOH as vehicle solution. Samples were equally loaded in 2 gels and membranes were cut to incubate anti-HIF1α and anti-β-actin antibodies, respectively. Each lane represents a different animal. (**c**) Full unedited gel for Fig. 1e. Liver protein extracts of mice treated with vehicle (glucose+NaOH) for 6 h or FG-4592 (20 mg/kg) for 6, 12 or 24 h. The membrane was cut in two to incubate anti-HIF1α and anti-β-actin antibodies, respectively. (**d**) Full unedited gel for Fig. 5i, showing femur protein extracts of mice on ND or HFD and treated with FG-4592 or vehicle. Two replicates were loaded per condition. Samples were equally loaded in 2 gels and the membranes were cut to incubate anti-AGEs and anti-Histone H3 antibodies, respectively.

Supplementary References

1 Montgomery, M. K. *et al.* Mouse strain-dependent variation in obesity and glucose homeostasis in response to high-fat feeding. *Diabetologia* **56**, 1129–1139 (2013). https://doi.org/10.1007/s00125-013-2846-8

2 Fajardo, R. J., Karim, L., Calley, V. I. & Bouxsein, M. L. A review of rodent models of type 2 diabetic skeletal fragility. *J Bone Miner Res* **29**, 1025–1040 (2014). https://doi.org/10.1002/jbmr.2210

3 Gautam, J. *et al.* Micro-architectural changes in cancellous bone differ in female and male C57BL/6 mice with high-fat diet-induced low bone mineral density. *Br J Nutr* **111**, 1811–1821 (2014). https://doi.org/10.1017/S0007114514000051

4 Pettersson, U. S., Walden, T. B., Carlsson, P. O., Jansson, L. & Phillipson, M. Female mice are protected against high-fat diet induced metabolic syndrome and increase the regulatory T cell population in adipose tissue. *PLoS One* **7**, e46057 (2012). https://doi.org/10.1371/journal.pone.0046057

5 Bohm, A. M. *et al.* Activation of Skeletal Stem and Progenitor Cells for Bone Regeneration Is Driven by PDGFRbeta Signaling. *Dev Cell* **51**, 236–254 e212 (2019). https://doi.org/10.1016/j.devcel.2019.08.013

6 Peredo, N. *et al.* Visualization and quantification of the stromal-vascular compartment in fetal or adult mouse bones: From sampling to high-resolution 3D image analysis. *STAR Protocols* **3** (2022). https://doi.org/10.1016/j.xpro.2022.101222

7 Berg, S. *et al.* ilastik: interactive machine learning for (bio)image analysis. *Nat Methods* **16**, 1226–1232 (2019). https://doi.org/10.1038/s41592-019-0582-9

8 Legland, D., Arganda-Carreras, I. & Andrey, P. MorphoLibJ: integrated library and plugins for mathematical morphology with ImageJ. *Bioinformatics* **32**, 3532–3534 (2016). https://doi.org/10.1093/bioinformatics/btw413

9 Dejaeger, M. *et al.* Integrin-linked kinase regulates bone formation by controlling cytoskeletal organization and modulating BMP and Wnt signaling in osteoprogenitors. *J Bone Miner Res* **32**, 2087–2102 (2017). https://doi.org/10.1002/jbmr.3190

10 Dirckx, N. *et al.* Vhl deletion in osteoblasts boosts cellular glycolysis and improves global glucose metabolism. *J Clin Invest* **128**, 1087–1105 (2018). https://doi.org/10.1172/JCI97794

11 Gardegaront, M., Farlay, D., Peyruchaud, O. & Follet, H. Automation of the Peak Fitting Method in Bone FTIR Microspectroscopy Spectrum Analysis: Human and Mice Bone Study. *Journal of Spectroscopy* **2018**, 1–11 (2018). https://doi.org/10.1155/2018/4131029

12 Farlay, D. *et al.* The ratio 1660/1690 cm(-1) measured by infrared microspectroscopy is not specific of enzymatic collagen cross-links in bone tissue. *PLoS One* **6**, e28736 (2011). https://doi.org/10.1371/journal.pone.0028736

13 Farlay, D., Panczer, G., Rey, C., Delmas, P. D. & Boivin, G. Mineral maturity and crystallinity index are distinct characteristics of bone mineral. *J Bone Miner Metab* **28**, 433–445 (2010). https://doi.org/10.1007/s00774-009-0146-7

14 Farlay, D. *et al.* Material and nanomechanical properties of bone structural units of cortical and trabecular iliac bone tissues from untreated postmenopausal osteoporotic women. *Bone Rep* **17**, 101623 (2022). https://doi.org/10.1016/j.bonr.2022.101623
